# Molecular, Pathological, Radiological, and Immune Profiling of Non-brainstem Pediatric High-Grade Glioma from the HERBY Phase II Randomized Trial

**DOI:** 10.1016/j.ccell.2018.04.004

**Published:** 2018-05-14

**Authors:** Alan Mackay, Anna Burford, Valeria Molinari, David T.W. Jones, Elisa Izquierdo, Jurriaan Brouwer-Visser, Felice Giangaspero, Christine Haberler, Torsten Pietsch, Thomas S. Jacques, Dominique Figarella-Branger, Daniel Rodriguez, Paul S. Morgan, Pichai Raman, Angela J. Waanders, Adam C. Resnick, Maura Massimino, Maria Luisa Garrè, Helen Smith, David Capper, Stefan M. Pfister, Thomas Würdinger, Rachel Tam, Josep Garcia, Meghna Das Thakur, Gilles Vassal, Jacques Grill, Tim Jaspan, Pascale Varlet, Chris Jones

**Affiliations:** 1 Division of Molecular Pathology, The Institute of Cancer Research, 15 Cotswold Road, Sutton, London, Surrey SM2 5NG, UK; 2 Division of Cancer Therapeutics, The Institute of Cancer Research, 15 Cotswold Road, Sutton, London, Surrey SM2 5NG, UK; 3 Hopp Children's Cancer Center at the NCT Heidelberg (KiTZ), Heidelberg, Germany; 4 Division of Paediatric Neuro-oncology, German Cancer Consortium (DKTK), German Cancer Research Center (DKFZ), Heidelberg, Germany; 5 Department of Pediatric Hematology and Oncology, Heidelberg University Hospital, Heidelberg, Germany; 6 Roche Innovation Center, New York, NY, USA; 7 Department of Radiology, Oncology and Anatomic-Pathology Sciences, Sapienza University, Rome, Italy; 8 IRCCS Neuromed, Pozzilli, Italy; 9 Institute of Neurology, Medical University of Vienna, Vienna, Austria; 10 Comprehensive Cancer Center, Medical University of Vienna, Vienna, Austria; 11 DGNN Brain Tumor Reference Center, Institute of Neuropathology, University of Bonn Medical Center, Bonn, Germany; 12 UCL Great Ormond Street Institute of Child Health, London, UK; 13 Department of Histopathology, Great Ormond Street Hospital for Children, London, UK; 14 Department of Pathology and Neuropathology, La Timone Hospital, Aix Marseille University, Marseille, France; 15 Nottingham University Hospitals, Nottingham, UK; 16 The Center for Data Driven Discovery in Biomedicine (D^3^b), Children's Hospital of Philadelphia, Philadelphia, PA, USA; 17 Division of Neurosurgery, Children's Hospital of Philadelphia, Philadelphia, PA, USA; 18 Division of Oncology, Children's Hospital of Philadelphia, Philadelphia, PA, USA; 19 Pediatric Oncology Unit, Fondazione IRCCS, Istituto Nazionale Tumori, Milan, Italy; 20 Centro di Neuro-Oncologia, Istituto Giannina Gaslini, Genoa, Italy; 21 F. Hoffmann-La Roche Ltd, Basel, Switzerland; 22 Charité – Universitätsmedizin Berlin, Corporate Member of Freie Universität Berlin, Humboldt-Universität zu Berlin, Berlin, Germany; 23 Berlin Institute of Health, Institute of Neuropathology, Berlin, Germany; 24 Department of Neuropathology, University Hospital Heidelberg and Clinical Cooperation Unit Neuropathology, German Cancer Consortium (DKTK), German Cancer Research Center (DKFZ), Heidelberg, Germany; 25 Department of Neurosurgery, Brain Tumor Center Amsterdam, VU Medical Center, Amsterdam, the Netherlands; 26 Genentech, South San Francisco, CA, USA; 27 Pediatric and Adolescent Oncology and Unite Mixte de Recherche 8203 du Centre National de la Recherche Scientifique, Gustave Roussy, Paris-Saclay University, Villejuif, France; 28 Sainte-Anne Hospital, Paris-Descartes University, Paris, France

**Keywords:** immune, CD8, MAPK, hypermutator, *H3F3A*, pediatric high-grade glioma

## Abstract

The HERBY trial was a phase II open-label, randomized, multicenter trial evaluating bevacizumab (BEV) in addition to temozolomide/radiotherapy in patients with newly diagnosed non-brainstem high-grade glioma (HGG) between the ages of 3 and 18 years. We carried out comprehensive molecular analysis integrated with pathology, radiology, and immune profiling. In *post-hoc* subgroup analysis, hypermutator tumors (mismatch repair deficiency and somatic *POLE*/*POLD1* mutations) and those biologically resembling pleomorphic xanthoastrocytoma ([PXA]-like, driven by *BRAF*_V600E or *NF1* mutation) had significantly more CD8^+^ tumor-infiltrating lymphocytes, and longer survival with the addition of BEV. Histone H3 subgroups (hemispheric G34R/V and midline K27M) had a worse outcome and were immune cold. Future clinical trials will need to take into account the diversity represented by the term “HGG” in the pediatric population.

## Highlights

• The HERBY trial tested the use of bevacizumab in pediatric non-brainstem HGG
• Parallel translational biology studies highlighted the diversity of the trial cohort
• Elevated levels of CD8^+^ T cells were seen in PXA-like and hypermutant tumors
• MAPK-associated immune signatures predicted response to bevacizumab

## Graphical Abstract

**Figure:**
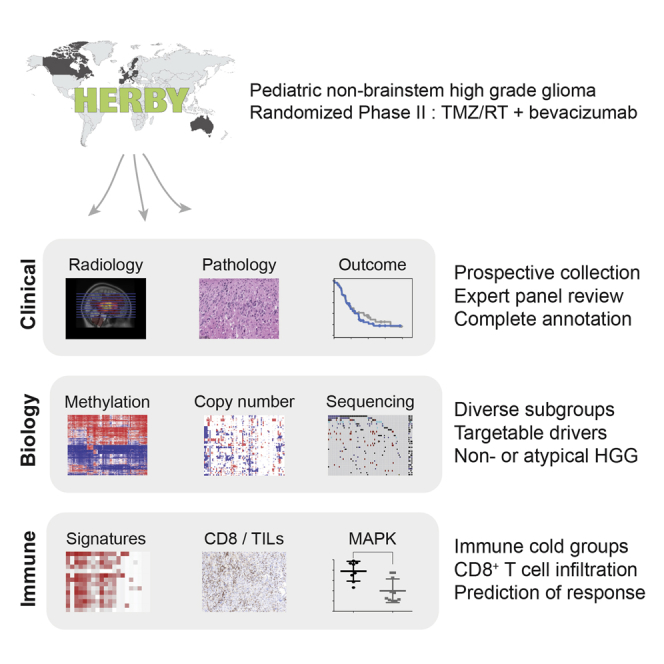

## Teaser

In a pediatric high-grade non-brainstem glioma cohort, Mackay et al. show that hypermutator tumors and those resembling pleomorphic xanthoastrocytoma are highly infiltrated by CD8^+^ lymphocytes and benefit from the addition of bevacizumab, whereas the histone H3 subgroups are immune cold and have a poor outcome.

## Significance

**We validate in the prospective clinical trial setting the biological and clinical diversity of pediatric high-grade glioma previously described in large retrospective series, underpinned by detailed pathological and radiological analysis. Although adding bevacizumab (BEV) to standard temozolomide/radiotherapy did not improve survival across the whole cohort, we identify disease subgroups with MAPK activation to harbor an enhanced CD8**^**+**^ **T cell immune response, which may derive benefit from the addition of BEV. If confirmed in another study, this would represent a useful predictive biomarker for this regimen in these tumors, and points the way for therapeutic strategies for subgroups of children with high-grade glioma.**

## Introduction

High-grade gliomas (HGGs) in children, like their adult counterparts, continue to have a bleak prognosis, with a median overall survival (OS) of 9–15 months (Cohen et al., 2011, Jones et al., 2016, Ostrom et al., 2015). Recent integrated molecular-profiling initiatives have shown that pediatric HGGs (pHGGs) are biologically distinct from their adult counterparts, with subgroups of the disease marked by recurrent mutations in genes encoding histone H3 variants having different age of incidence, anatomical location, clinical outcome, and a range of biological parameters (Jones and Baker, 2014, Mackay et al., 2017, Paugh et al., 2010, Schwartzentruber et al., 2012, Sturm et al., 2012, Wu et al., 2012, Wu et al., 2014). Histone wild-type (WT) tumors have widely differing mutational burdens, ranging from infant cases (<3 years) driven by single gene fusion events through to patients with biallelic mismatch repair deficiency harboring some of the highest mutational rates in human cancer (Jones and Baker, 2014, Mackay et al., 2017, Shlien et al., 2015, Wu et al., 2014).

The rapid advances in our understanding of pHGGs have come predominantly from the accumulation of numerous disparate retrospective collections, a reflection of the rarity of the disease. Clinical trial cohorts with ancillary biomarker analyses have been relatively limited in their scope, and historically have focused on single-marker analyses. These include the Children's Oncology Group ACNS0126 (radiotherapy [RT]/temozolomide [TMZ]) (Cohen et al., 2011) and ACNS0423 (RT/TMZ followed by TMZ and lomustine) (Jakacki et al., 2016) studies, which report on the frequency and clinical correlations of O6-methylguanine-DNA methyltransferase (MGMT) expression (ACNS0126) (Jakacki et al., 2016, Pollack et al., 2006), *IDH1* mutation (ACNS0423) (Pollack et al., 2011), as well as phosphorylated Akt expression (Pollack et al., 2010a) and microsatellite instability (both) (Pollack et al., 2010b). The CCG-945 study (“8 in 1” chemotherapy) (Finlay et al., 1995) reported on the prognostic significance of p53 expression/mutation (Pollack et al., 2002), in addition to the presence/absence of 1p19q co-deletion (Pollack et al., 2003b).

This last study (Pollack et al., 2003b) also highlighted the critical importance of pathological review in the diagnosis of pHGG, and subsequent interpretation of clinical trial results (Gilles et al., 2008, Pollack et al., 2003a), particularly in midline locations (Eisenstat et al., 2015). It has subsequently become clear that numerous histological subtypes of HGG can harbor distinct genetic drivers and have considerably better clinical outcomes, such as *BRAF*_V600E mutations in epithelioid glioblastoma (GBM), anaplastic ganglioglioma, and anaplastic pleomorphic xanthoastrocytoma (PXA) (Hatae et al., 2016); in the latter two categories, this mutation is also found in lower-grade entities lacking obvious anaplasia. Additional histone WT cases of otherwise uncontroversial HGGs have been found to be biologically and clinically more similar to several types of low-grade glioma (LGG) and PXA (Korshunov et al., 2015), highlighting the importance of an integrated diagnosis combining molecular and histological features.

The HERBY trial (study BO25041; clinicaltrials.gov NCT01390948) was a phase II, open-label, randomized, multicenter, comparator study of the addition of the anti-angiogenic agent bevacizumab (BEV) to RT and TMZ in patients between the ages of 3 and 18 years with newly diagnosed non-brainstem HGG (Grill et al., 2018). Central confirmation of HGG diagnosis was mandatory before randomization, followed by consensus review by five independent expert neuropathologists. Real-time panel radiological assessment was also incorporated. An exploratory endpoint of the study was to establish a biospecimen repository for correlative molecular profiling. In addition to its role in tumor angiogenesis, vascular endothelial growth factor (VEGF) restricts immune cell activity, and BEV has been demonstrated to facilitate recruitment of T cells to infiltrate tumors (Wallin et al., 2016), as well as increase the ratio of CD8^+^CD3^+^ T cells in adult GBM specimens (Scholz et al., 2016). We therefore also sought to explore the immune profile of cases within the HERBY cohort.

## Results

### The Translational Research Cohort Is Representative of the Whole Clinical Trial Population

**Figure 1:**
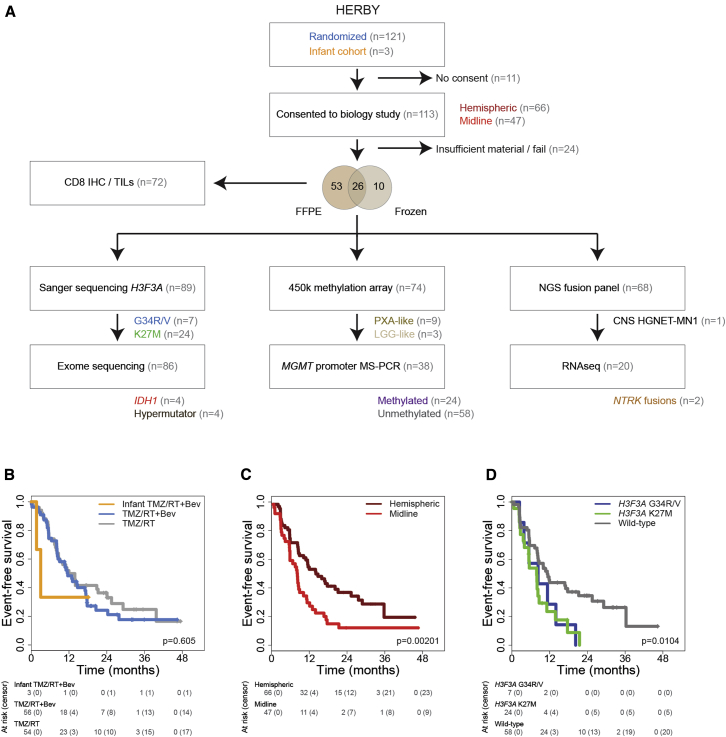
Sample Cohort (A) Flow diagram indicating total HERBY trial cohort (n = 121 randomized plus 3 infants), those patients consenting to the biology study (n = 113) for whom sufficient formalin-fixed paraffin-embedded (FFPE) or frozen tumor was available (n = 89), and the respective molecular analyses undertaken. (B–D) Kaplan-Meier plots of event-free survival of cases (y axis) separated by treatment arm (B), anatomical location (C), or *H3F3A* status (D), with time given in months (x axis) and the overall p value provided, calculated by the log rank test. Individual pairwise comparisons are provided in the text. See also Table S1.

The total HERBY cohort comprised 121 randomized patients at diagnosis (3–18 years) plus 3 infant cases (<3 years) at relapse. Of these, 113 patients consented to the translational research program (Table S1). Tumor tissue was collected from either resection (n = 93) or biopsy (n = 20), although 24 cases failed to provide sufficient quantity or quality of sample for molecular analysis. For the remaining 89 cases, material was available in the form of either fresh-frozen material (n = 36), formalin-fixed paraffin-embedded pathology specimens (n = 79), or both (n = 26). These were subjected to Sanger sequencing for *H3F3A* (n = 89), exome sequencing (n = 86), Illumina 450k methylation BeadChip profiling (n = 74), CD8 immunohistochemistry (n = 70), methylation-specific PCR for *MGMT* promoter methylation (n = 36), a capture-based sequencing panel for common fusion gene detection (n = 68), and RNA sequencing (RNA-seq) (n = 20) (Figure 1A).

The translational research cohort, representing a subset (91%) of the randomized trial, displayed equivalent clinical characteristics to the full dataset (Grill et al., 2018), with no difference in the primary endpoint of 1-year event-free survival (EFS) with the addition of BEV to the standard therapy of TMZ and RT (median 12.0 versus 8.3 months, p = 0.372, log rank test), with an additional small (n = 3) infant cohort treated with BEV at relapse (Figure 1B). The cohort contained 66 (58%) hemispheric and 47 (42%) non-brainstem midline tumors, with the latter location conferring a significantly shorter EFS (median 8.0 versus 14.7 months, p = 0.00201, log rank test) (Figure 1C). Histone mutation status was a significant predictor of worse prognosis compared with WT (median EFS = 11.3 months) for *H3F3A*_K27M (24/89, 27%; median EFS = 7.9 months; p = 0.0063, log rank test) and also trended toward shorter survival for *H3F3A*_G34R/V (7/89, 8%; median EFS = 8.3 months; p = 0.096, log rank test) (Figure 1D).

### Integrated Molecular Analysis Defines the Major (Epi)genomic Alterations in pHGG

**Figure 2:**
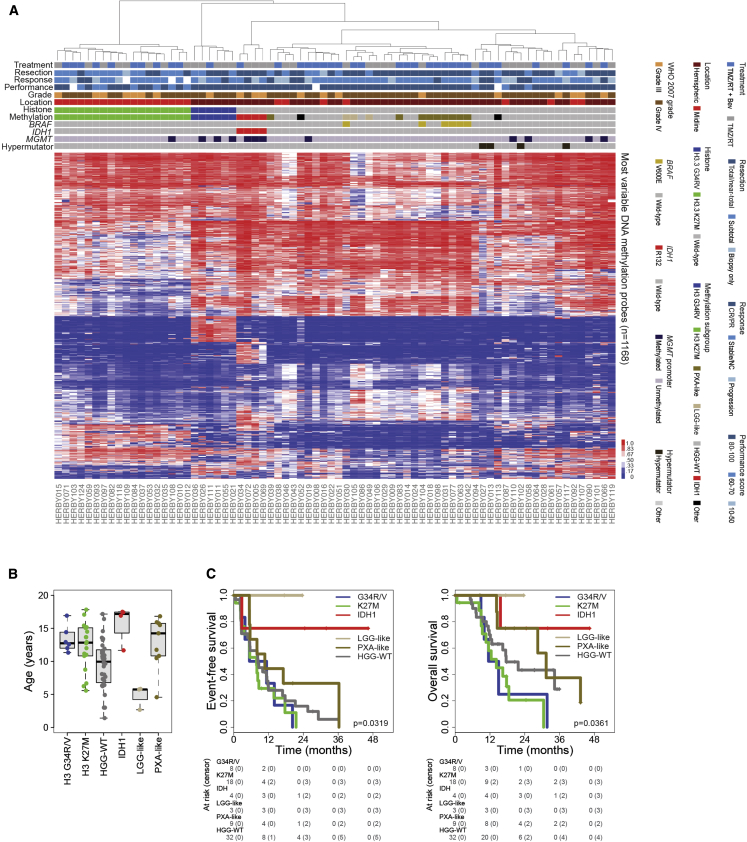
Methylation-Based Subclassification (A) Heatmap representation of β values for 74 samples profiled on the Illumina 450k BeadChip platform (red, high; blue, low). Samples are arranged in columns clustered by probes with the largest median absolute deviation across the 10k predictor subset of probes. Clinicopathological and molecular annotations are provided as bars according to the included key. CR/PR, complete response or partial response; Stable/NC, stable disease or no change. (B) Boxplot showing age at diagnosis of included cases, separated by methylation subclass. The thick line within the box is the median, the lower and upper limits of the boxes represent the first and third quartiles, and the whiskers 1.5× the interquartile range. (C) Kaplan-Meier plot of event-free and overall survival of cases (y axis) separated by methylation subclass, time given in months (x axis) and overall p value calculated by the log rank test. See also Figure S1 and Table S2.

We used the Heidelberg brain tumor classifier on Illumina 450k methylation array data to assign a molecular subgroup to each of 74 samples for which such data were available (Table S2). After excluding low-scoring assignments (<0.2), we used a simplified system to classify tumors as either H3K27M (n = 18), H3G34R/V (n = 6), or IDH1 (n = 4) (integrating gene mutation data in low-scoring cases); as resembling PXA-like (n = 9) or other LGG-like (n = 3); and aggregating the remaining tumors (HGG-WT, n = 34) (Figure 2A). IDH1 tumors represented the oldest patients (median = 17.2 years, others = 11.2, p = 0.0107, t test), with LGG-like representing the youngest category (median = 5.7 years, p = 0.0098, t test) (Figure 2B). These two subgroups each had significantly better outcome in terms of EFS (p = 0.0281 and p = 0.0386, log rank test), although not OS (p = 0.0935 and p = 0.129, log rank test) (Figure 2C), when compared individually to the remaining tumors. The PXA-like showed a trend toward longer OS (p = 0.0867, log rank test) compared with the rest. When IDH1, PXA-like, and LGG-like tumors were excluded from the analysis, the significant differences between histone mutant and HGG-WT groups were absent (H3K27M, p = 0.257 EFS and p = 0.0746 OS; H3G34R/V, p = 0.552 EFS and p = 0.116 OS, log rank test). Twelve out of 78 (15%) samples harbored a methylated *MGMT* promoter, although this was largely restricted to the H3G34R/V (n = 3, p = 0.0249, Fishers exact test) and IDH1 (n = 3, p = 0.0062, Fisher’s exact test) subgroups (Figure S1A), and was not significantly associated with survival (Figure S1B) in these uniformly TMZ-treated patients.

**Figure 3:**
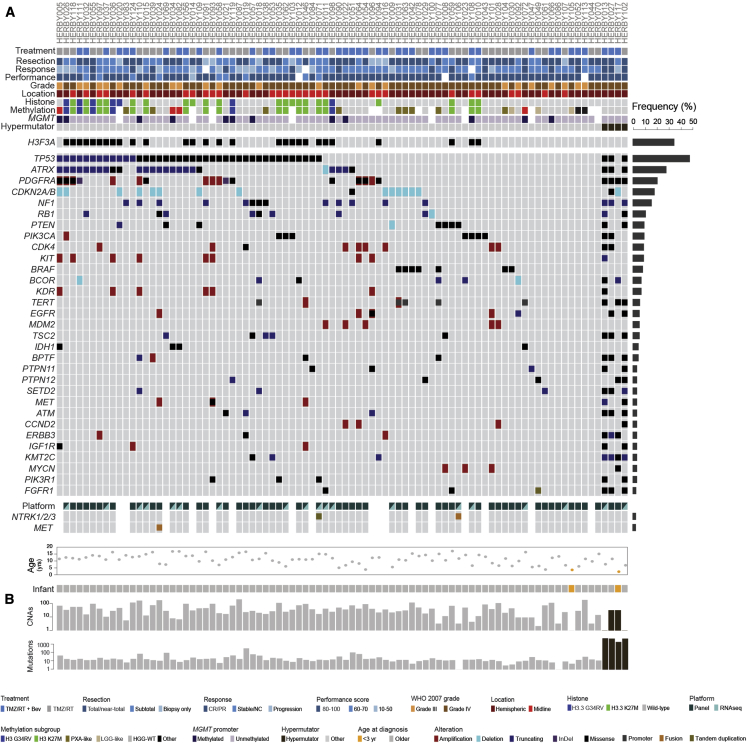
Somatic Mutations (A) Oncoprint representation of an integrated annotation of somatic mutations and DNA copy-number changes for the 30 most frequently altered genes in 86 samples (n ≥ 3, frequency barplot on the right, excluding hypermutator cases). Selected common fusion events are also shown where available. Samples are arranged in columns with genes labeled along rows. (B) Barplots are provided on a log_10_ scale for numbers of copy-number aberrations and somatic mutations per case. Clinicopathological and molecular annotations are provided as bars according to the included key. CR/PR, complete response or partial response; Stable/NC, stable disease or no change. See also Figures S2 and S3 and Table S3.

We used a 450k methylation array and exome-sequencing coverage to derive DNA copy-number profiles for 86 pHGG (Figure S2A), including focal amplifications/deletions, as well as whole-arm chromosomal gains/losses (Table S3). Taken with the somatic single-nucleotide variants (SNVs) and small insertion/deletions from whole-exome sequencing (Table S3), and candidate gene fusion events from capture-based panel sequencing (n = 68) and RNA-seq (n = 20) (Table S3), we derived an integrated map of genetic alterations across the translational research cohort (Figure 3A). The most common alteration was *TP53* mutation (39/82, 48%), followed by *ATRX* deletion/mutation (25/82, 30%), *PDGFRA* amplification/mutation (17/82, 21%), and *CDKN2A/B* deletion (15/82, 18%). Additional recurrent alterations in receptor tyrosine kinases (*EGFR, MET, ERBB3, IGF1R,* and *NTRK2*), phosphatidylinositol 3-kinase (PI3K)/mammalian target of rapamycin (*PTEN, PIK3CA, TSC2,* and *PIK3R1*), and MAP-kinase (*NF1, BRAF, PTPN11,* and *PTPN12*) pathways were common, as were amplifications/mutations in various genes associated with cell-cycle regulation (*RB1, CDK4, MDM2,* and *CCND2*). Taking a minimum variant allele frequency of 5% as a threshold, the median number of somatic mutations per sample was 15 (range = 0–337) (Figure 3B), with the exception of four cases for whom there were more than 100-fold more, and were excluded from gene-level counts.

### pHGGs Comprise a Diverse Set of Biological and Clinicopathological Subgroups

Two cases were highlighted from the methylation subgrouping as potentially representing non-HGG entities. One case classifying as CNS neuroblastoma with FOXR2 activation (CNS NB-FOXR2, methylation classifier score = 0.617), was found to have no evidence of *FOXR2* alterations. A further case, a compact and necrotic tumor with perivascular radiating arrangements (Figure S2B), displayed a methylation classifier score strongly indicative of a high-grade neuroepithelial tumor with *MN1* alteration (CNS HGNET-MN1, methylation classifier score = 0.713) (Figure S2C). We identified a candidate alteration in this case fusing exon 1 of *MN1* (22q12.1) to exon 3 of *CARD6* (5p13.1) (Figure S2D), and thus appears most likely to fall into this categorization.

Three cases classified more closely to either pilocytic astrocytoma (n = 2) or desmoplastic infantile ganglioglioma (DIG) (n = 1) by 450k methylation profiling. The first two harbored mitogen-activated protein kinase (MAPK) dysregulation in the form of either *BRAF*_V600E or intragenic *FGFR1* duplication (Figure S2E). Histologically, after Pathology Committee re-review, no piloid features were seen, and anaplastic features were evident (Figure S2F). The DIG-like case was found in the infant cohort (2.7 years). None of these three patients died during the course of follow-up, and, although numbers are small, were all found in the right frontal and temporo-parietal lobes with central predominance (Figure S2G).

There were four cases with *IDH1* hotspot mutations (Figure S3A). Three were classified as WHO-grade III anaplastic astrocytoma (AA), *IDH1*_R132-positive by immunohistochemistry, with concurrent *TP53* and *ATRX* mutations. The remaining case was originally classified as a mixed oligo-astrocytoma, which, by virtue of the presence of *IDH1*_R132 and *TERT* promoter mutation (C228T), as well as copy-number loss of chromosomes 1p and 19q, would be described as an oligodendroglioma according to WHO 2016 (Figure S3B). Across the whole cohort, *IDH1* mutation conferred a significantly longer EFS (p = 0.0398, log rank test), although not OS (p = 0.110, log rank test) (Figure S3C), and were restricted to the frontal lobes (Figure S3D).

After excluding *IDH1* mutant cases, the remaining *H3F3A* and *BRAF* WT cases (n = 38) represented a heterogeneous mix of genomic profiles, with recurrent deletions/mutations in the common pHGG tumor suppressor genes *TP53* (n = 11), *ATRX* (n = 5), *CDKN2A/B* (n = 7), *NF1* (n = 8), *RB1* (n = 7), and *PTEN* (n = 5), but also with an enrichment of gene amplifications in *PDGFRA* (n = 5, with *KIT* and *KDR*, n = 4), *CDK4* (n = 7, with *MDM2*, n = 4), *EGFR* (n = 4), *MET* (n = 2), *CCND2* (n = 3), and *MYCN* (n = 3) (Figure S3E). The most common methylation subclass in these cases was designated GBM_RTK_MYCN (n = 6); however, it included only one of those with *MYCN* amplification, and with no other common amplifications or mutations. Seven cases harbored none of the recurrently altered genes previously described in pHGG, and clearly represent a subgroup that warrants further investigation. Together, these cases had bilateral hemispheric distribution with predominant deep right cerebral localization (Figure S3F).

### *H3F3A* Mutation Confers Poor Prognosis for Both K27M and G34R/V Substitutions

**Figure 4:**
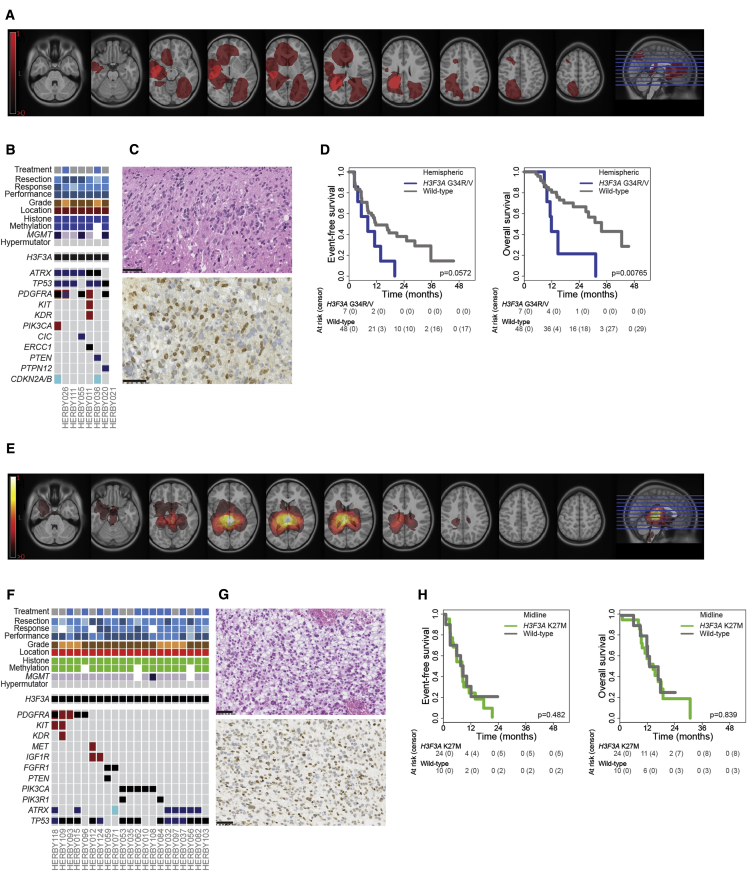
*H3F3A* Mutant Subgroups (A–D) *H3F3A*_G34R/V. (A) Radiological tumor lesion map of 7 *H3F3A*_G34R/V cases. Brighter colored pixels indicate a higher probability of tumor incidence. (B) Integrated annotation of somatic mutations and DNA copy-number changes in *H3F3A*_G34R/V cases. Clinicopathological and molecular annotations are provided as bars according to the included key in Figure 3. (C) H&E (top) and immunohistochemistry (bottom) directed against H3.3G34R for HERBY020. Scale bars, 50 μm. (D) Kaplan-Meier plot of event-free and overall survival of 55 hemispheric cases (y axis) separated by *H3F3A* status, time given in months (x axis), p value calculated by the log rank test. (E–H) *H3F3A*_K27M. (E) Radiological tumor lesion map of 21 *H3F3A*_K27M cases. Brighter colored pixels indicate a higher probability of tumor incidence. (F) Integrated data from *H3F3A*_K27M cases. Clinicopathological and molecular annotations are provided as bars according to the included key in Figure 3. (G) H&E (top) and immunohistochemistry (bottom) directed against H3.3K27M for HERBY015. Scale bars 50 μm. (H) Kaplan-Meier plot of event-free and overall survival of 34 midline cases (y axis) separated by *H3F3A* status, time given in months (x axis), p value calculated by the log rank test. See also Figure S4.

Histone mutations have been shown to be present in approximately half of all pHGGs (Mackay et al., 2017), with a clear negative impact on survival for K27M (Karremann et al., 2018, Khuong-Quang et al., 2012, Mackay et al., 2017), although the situation is less clear for G34R/V mutations (Bjerke et al., 2013, Korshunov et al., 2015, Mackay et al., 2017). *H3F3A*_G34R/V mutant tumors had a tendency to being diffusely infiltrative with predominant deep left temporo-parietal involvement (Figure 4A). There were seven cases with *H3F3A*_G34 substitutions (six G34R and one G34V), with six out of seven (86%) cases additionally harboring *TP53* and/or *ATRX* mutations (five out of seven, 71% both), while five out of seven (71%) also contained *PDGFRA* amplification and/or mutation (Figure 4B). There were no other recurrent mutations, although isolated instances of mutations in PI3K signaling (*PIK3CA* and *PTEN*) and DNA repair (*ERCC1*) were observed (Table S3). Histologically, there were four GBM, two AA with multinucleated cells, and one HGG, not otherwise specified (Figure 4C). Tumors were Olig2 negative (7/7) with strong nuclear accumulation of p53 (6/7). Across all tumors within this hemispheric subgroup, patients harboring these mutations trended toward a shorter EFS (median = 8.3 months; p = 0.0572, log rank test) and had a significantly shorter OS (median = 12.0 months; p = 0.00765, log rank test) (Figure 4D), although this association was lost when IDH1, PXA-like, and LGG-like tumors were excluded (p = 0.440 EFS and p = 0.139 OS, log rank test) (Figure S4A).

By contrast, K27M substitutions were restricted to midline regions (n = 24). Two patients had distinct, separate lesions in the thalamus and hypothalamus, while the remaining had central thalamic, midbrain, or cerebellar localization (Figure 4E). Fifteen out of 21 (71%) exome-sequenced cases carried additional amplifications/mutations in the receptor-tyrosine kinase (RTK)-PI3K pathway across a range of genes (*PDGFRA, MET, IGF1R, FGFR1, PTEN, PIK3CA,* and *PIK3R1*), with five out of six of the remaining tumors harboring *ATRX* mutation (Figure 4F; Table S3). There was strong immunoreactivity for H3K27M in 12/12 cases tested (Figure 4G). Although conferring a worse prognosis across the whole cohort (above), within midline locations there was no association with either EFS (median = 7.9 months; p = 0.482, log rank test) or OS (median = 14.2 months; p = 0.839, log rank test) (Figure 4H), nor any prognostic value for WHO-grade in K27M tumors (p = 0.646 EFS and p = 0.762 OS, log rank test) (Figure S4B).

### Immune-Positive Subgroups Are Associated with MAPK and Benefit from the Addition of BEV

**Figure 5:**
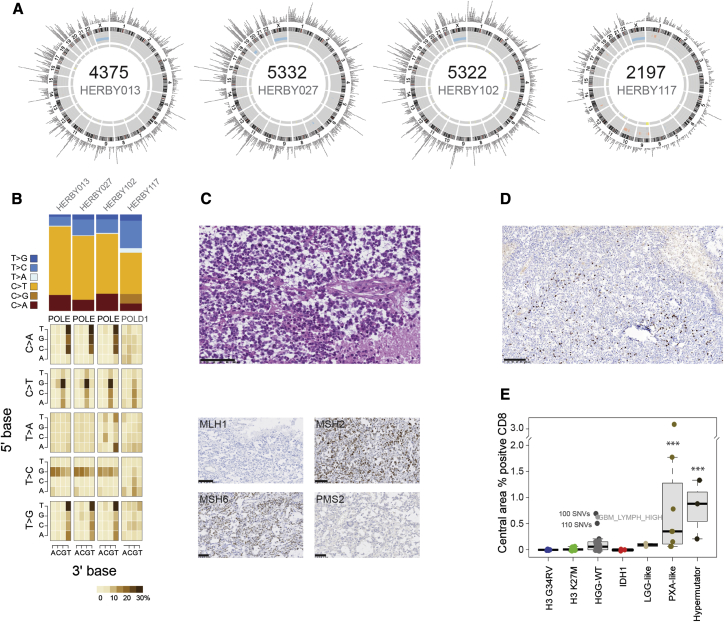
Hypermutator Cases (A) Circos plots for four hypermutator cases. In each case, plots provide somatic SNVs and insertion/deletions on the outer ring, DNA copy-number changes (dark red, amplification; red, gain; dark blue, deletion; blue, loss), and loss of heterozygosity (yellow) on the inner rings, and intra- (orange) and inter- (blue) chromosomal translocations inside the circle. (B) Mutation signatures. Top: simple stacked barplot representation of the proportion of mutation types observed in individual hypermutator cases and the remaining accumulated dataset. Base changes given in the key. Bottom: mutation context given for each of the 96 mutated trinucleotides, represented by heatmap. The base located 5′ to each mutated base is shown on the vertical axis, and the 3′ base is on the horizontal axis. (C) H&E (top) and protein expression of mismatch repair proteins (bottom panel, clockwise from top left: MLH1, MSH2, MSH6, and PMS2) as assessed by immunohistochemistry in a glioblastoma with 5,322 somatic mutations (HERBY102). Scale bars, 100 μm (H&E, MLH1, MSH2) or 50 μm (MSH6, PMS2). (D) CD8 expression in T cells by immunohistochemistry in HERBY102. Scale bar, 100 μm. (E) Boxplot of percentage of CD8^+^ cells in the central tumor region separated by subgroup. The thick line within the box is the median, the lower and upper limits of the boxes represent the first and third quartiles, and the whiskers 1.5× the interquartile range. ^∗∗∗^p < 0.0001, t test. See also Figure S5 and Table S4.

pHGGs with a high mutational burden had previously been described to have an elevated neoantigen load and a pronounced immune response (Bouffet et al., 2016). Four cases were classed as hypermutator, with a median somatic mutation count of 4,848 (range = 2,197–5,332; mutation rate 160–240 mutations/Mb) (Figure 5A, Table S1). Mutation signature analysis showed predominantly C > T transitions and hotspot somatic *POLE* mutations in three cases, and a somatic *POLD1* mutation in the fourth (Figure 5B). They were all categorized as GBM, and, in one case, the presumed mismatch repair deficiency was demonstrated by clear loss of MLH1 expression by immunohistochemistry (Figure 5C). This specimen had a heterogeneous immune phenotype, with a relatively high percentage of CD8^+^ cells in the central area, and in three or four thin perivascular cuffs (Figure 5D; Table S4). These hypermutator cases had the highest percentage of CD8^+^ cells (p < 0.0001 t test versus rest excluding PXA-like) (Figure 5E). Notably, PXA-like tumors were also significantly enriched for CD8^+^ cells (p < 0.0001 t test versus rest excluding hypermutator). Of three HGG-WT cases with relatively high immune infiltrate, two had elevated somatic SNV counts (100–110 per case), while the third scored highly for the GBM_LYMPH_HI subgroup by methylation profiling. A formal histological assessment of tumor-infiltrating lymphocytes (TILs) confirmed the highest-scoring categories (lymphocytes scattered among tumor cells/in more than 50% of the tumor) to be almost exclusively present in hypermutator and PXA-like subgroups (Figure S5A; Table S4), as were those cases formally classified as an inflamed immune phenotype (Figure S5B; Table S4). Histone mutant tumors were notably immune cold as defined by a lack of CD8 immunoreactivity and an absence of TILs.

**Figure 6:**
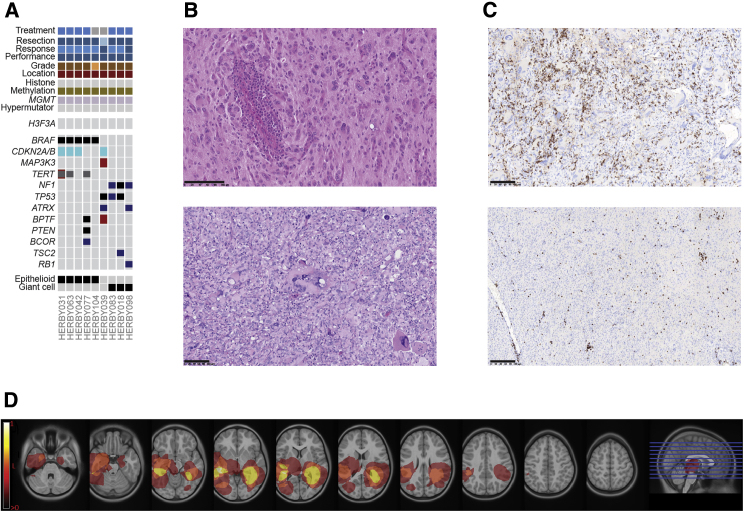
PXA-like Tumors (A) Integrated annotation of somatic mutations and DNA copy-number changes in nine samples classifying as PXA-like. Clinicopathological and molecular annotations are provided as bars according to the included key in Figure 3. (B) H&E staining of an epithelioid (top, HERBY104) and giant cell (bottom, HERBY098) variant of glioblastoma. Scale bars, 100 μm. (C) CD8 immunohistochemistry for the same cases as (B), marking CD8^+^ T cells. Scale bars, 100 μm. (D) Radiological tumor lesion map of PXA-like cases. Brighter colored pixels indicate a higher probability of tumor incidence.

Nine cases classified by 450k methylation profiling as more similar to PXAs than HGGs. Five out of nine (55%) harbored *BRAF*_V600E mutations, with three out of five (60%) also containing *CDKN2A/B* deletions and/or *TERT* amplification or promoter mutation (C250T) (Figure 6A). Three of four of the remaining cases were instead found with somatic *NF1* mutation, often in concert with *TP53* (three out of four) and/or *ATRX* mutation (two out of four). Upon histological re-review according to WHO 2016 guidelines by the HERBY Pathology Committee, *BRAF*_V600E, cases were all found to comprise the epithelioid variant of grade IV GBM, while the *NF1* cases were all classified as the giant cell variant (Figure 6B). PXA-like tumors had a high degree of immune infiltrate, with cases exhibiting several perivascular cuffs of more than three layers of CD8^+^ T cells, which were also scattered among tumor cells in more than 50% of the specimen (Figure 6C). They were found bilaterally restricted to temporo-parietal regions with a medial hemispheric predominance (Figure 6D).

**Figure 7:**
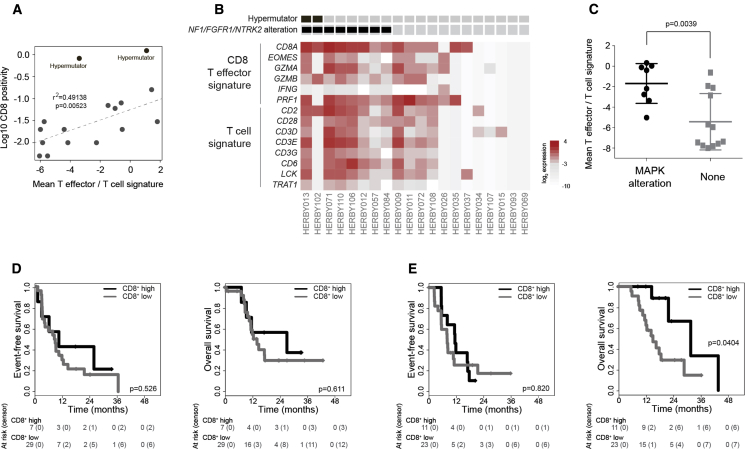
Immune Signatures and Response to BEV (A) Mean T effector/T cell gene expression values (plotted as log_2_, x axis) were correlated with CD8 immunoreactivity (plotted as log_10_, y axis) for 18 cases. Two cases were scored as 0% by immunohistochemistry and were not plotted (expression values −7.37 and −7.58). (B) Gene expression signatures for CD8 T effector and T cells plotted as a heatmap from 20 cases with RNA-seq data. Hypermutator cases and those with MAPK alterations including *NF1*, *FGFR1*, or *NTRK2* are annotated. (C) Plot of T effector/T cell gene expression values in MAPK-altered samples compared with those without. Horizontal bar represents the mean, error bars the SD. (D) Kaplan-Meier plot of event-free and overall survival of 36 cases (y axis) treated with TMZ/RT, separated by levels of CD8^+^ T cells, time given in months (x axis), and p value calculated by the log rank test. (E) Kaplan-Meier plot of event-free and overall survival of 34 cases (y axis) treated with TMZ/RT plus BEV, separated by levels of CD8^+^ T cells, time given in months (x axis), and p value calculated by the log rank test. See also Figures S6 and S7 and Table S5.

Gene expression data from RNA-seq were available for a subset of samples, in which a CD8 T effector/T cell signature was found to correlate with CD8 positivity by immunohistochemistry (r^2^ = 0.49138, p = 0.00523), with two hypermutator cases as outliers (Figure 7A). Although no PXA-like or *BRAF*_V600E mutant cases were included in this subset, these signatures were particularly evident in cases with predicted MAPK pathway-activating alterations in *NF1* (truncating frameshift/nonsense, disrupting translocation or predicted damaging missense), *NTRK2* (translocation or tandem duplication of kinase domains), and *FGFR1* (known activating hotspot mutation) (Figure 7B). Gene set enrichment analysis (GSEA) showed multiple enrichments for gene sets associated with T cell signaling and the immune response (e.g., PID_CD8_TCR_ DOWNSTREAM_PATHWAY, enrichment score = 0.594, nominal p = 0.0020; KEGG_T_CELL_RECEPTOR_SIGNALING_PATHWAY, enrichment score = 0.532, nominal p = 0.0297) and inflammatory-related MAPK signaling (e.g., ST_JNK_MAPK_PATHWAY, enrichment score = 0.466, nominal p = 0.0332; REACTOME_GRB2_SOS_PROVIDES_ LINKAGE_TO_MAPK_SIGNALING_FOR_INTERGRINS, enrichment score = 0.581, nominal p = 0.0239) (Table S5), with mean T effector/T cell signature significantly higher than in cases without MAPK alterations (p = 0.0039, t test) (Figure 7C). This was validated in a restricted cohort of n = 59 patients from our retrospective analysis designed to approximate the HERBY cohort (i.e., non-brainstem pHGGs aged 3–18 years), in which we observed a significantly elevated T effector/T cell gene expression signature in MAPK-altered samples (p = 0.0018, t test) (Figures S6A and S6B), further demonstrated by GSEA (e.g., PID_CD8_TCR_ DOWNSTREAM_PATHWAY, enrichment score = 0.551, nominal p = 0.0099; BIOCARTA_TCR_PATHWAY enrichment score = 0.520, nominal p = 0.0305) (Table S5).

We also explored other immune-related gene expression signatures in the RNA-seq data, and noted a trend toward an elevated macrophage M2 response in MAPK-altered tumors (p = 0.0810, t test) (Figures S6C and S6D). We performed CD68 staining for a limited number of cases with a histologically defined immune response (n = 11), and found a heterogeneously distributed tumor-associated macrophages (TAM) component, comprising either TAM-free tumoral areas, or rich TAM areas especially around necrotic foci or associated with perivascular lymphocytes (Figure S6E). *BRAF*_V600E cases had CD68^+^ TAM more diffusely intermingled with tumor cells (Figure S6E). Although the presence of macrophage infiltration has previously been reported in diffuse intrinsic pontine glioma (Caretti et al., 2014), we observed no association of the M2 gene expression signature with K27M midline tumors in our cohort (p = 0.965).

With the primary efficacy analysis failing to demonstrate survival differences between the two arms, we explored whether the molecular-profiling data could identify subsets of patients who may have benefited from the addition of BEV to the standard chemoradiotherapy. We performed a univariate Cox regression analysis on methylation-based molecular subgroups, as well as individual gene-level alterations, and found none which had significant difference in outcome between investigational arms (Figure S7A), although *NF1*, *PDGFRA*, and *TP53* were adverse prognostic markers across the whole cohort (Figures S7B–S7E). Notably, however, while not predictive in the TMZ/RT arm (Figure 7D), the presence of high levels of CD8^+^ T cells within the central tumor area conferred a significantly better OS in children receiving the addition of BEV (p = 0.0404, log rank test) (Figure 7E). Fitting a Cox interaction model (with proportional hazards confirmed by calculating Schoenfeld residuals, p = 0.268), high CD8 levels trended toward predictivity of response to TMZ/RT + BEV, with a hazard ratio of 0.360 (p = 0.066). In keeping with the strong subgroup associations described, 17/18 cases with high levels of CD8^+^ T cells are hemispheric (representing 38.6% cases in this location). Within hemispheric tumors only, there is a significant interaction between high CD8 and BEV (hazard ratio [HR] = 0.251, p = 0.024).

## Discussion

The HERBY trial opened in October 2011, prior to the discovery of histone gene mutations in 2012 (Schwartzentruber et al., 2012), and the more extensive genome sequencing published in 2014 (Wu et al., 2014), and there were no molecular markers incorporated into trial design. The present correlative biology study demonstrates the extent of the heterogeneous population of tumors, with widely differing biological drivers and clinicopathological features that were included in the cohort. An important example is the four adolescent patients with *IDH1* mutation, who had a significantly better clinical outcome, and may now be thought of as the lower age limit of an adult subgroup rather than pHGGs. Such patients will likely be included in future *IDH1*-focussed trials across adult and pediatric oncology services in order to maximize the possibility of positive trial outcomes. Similarly, within the HERBY cohort were seven patients with *BRAF*_V600E mutations, who would now be candidates for upfront trials of targeted inhibitors such as vemurafenib (Bautista et al., 2014, Robinson et al., 2014), and four hypermutator patients, with somatic *POLE/POLD1* mutations and likely harboring biallelic mismatch repair deficiency syndrome, who may benefit from immune checkpoint inhibitors such as nivolumab (Bouffet et al., 2016). In both instances, early clinical data show remarkably promising results and there would be little justification in their continued inclusion in catch-all HGG trials.

Notably, both hypermutator cases, and those biologically resembling PXAs, the latter of which harbored either *BRAF-* or *NF1*-driven MAP-kinase alterations, were found to be the most immunogenic in terms of CD8^+^ T cells/TILs, including a significantly elevated CD8 effector T cell gene expression signature. This is important given reports in adult GBM of an immunosuppressive phenotype associated with an elevated CD8^+^ regulatory T cell immune infiltrate (Kmiecik et al., 2013). The MAPK-altered hypermutator and PXA-like pediatric cases in the present cohort were found to benefit from the addition of BEV to TMZ/RT as regard to overall, although not EFS, the first and only such predictive biomarker identified in this patient population. The immunomodulatory effects of anti-angiogenic therapies have been previously demonstrated in other cancer types (Elamin et al., 2015), with VEGF-A shown to play an important role in the induction of an immunosuppressive environment (Gabrilovich et al., 1996), through increasing PD-L1 and other inhibitory checkpoints involved in CD8^+^ T cell exhaustion.

An important implication of these observations is the possibility of overcoming resistance to BRAF/MEK-targeting therapies by combining them with BEV and/or other immune therapies in these subgroups, as has been suggested in melanoma (Hu-Lieskovan et al., 2015). Such a strategy is, however, complicated by the diverse roles on T cell function played by MAPK signaling. Although involved in the regulation of T cell proliferation and survival (D'Souza et al., 2008), selective BRAF inhibitors have been shown to increase CD8^+^ lymphocytes in human metastatic melanoma models (Wilmott et al., 2012). Crucially, although MEK inhibition has been demonstrated to block naive CD8^+^ T cell priming in a colon cancer model, the number of CD8^+^ effector T cells within the tumor were increased, and could potentiate immune checkpoint therapy (Ebert et al., 2016). A limitation of the present study is the small numbers in this *post-hoc* analysis, and the benefit that patients with these biological subgroups may derive from an immunomodulatory mechanism of BEV would need to be tested in the prospective setting. This is a challenge given that these patients represent approximately 10%–15% of an already rare disease; however, international collaborative trials groups (such as those in HERBY represent), already recruit hypermutator and MAPK-altered HGGs in this population for appropriately targeted therapies (NCT02992964, NCT02684058). Equally importantly, the histone H3 mutant subgroups, which represent a substantial proportion of patients in this age group (Mackay et al., 2017) were found to be very poorly immunogenic, confirming a previous study in resectable malignant brainstem gliomas in children and adults with K27M mutations (Zhang et al., 2017), and further negating the likelihood of clinical response to such therapies.

A key observation is the high prevalence of tumors occurring outside the cerebral hemispheres harboring histone mutations, included in the most recent 2016 WHO classification system as a separate entity called diffuse midline glioma with H3K27M mutation. These represented 27% of the assessed population, and had a particularly poor outcome, as did histone WT midline cases. These tumors only rarely have *MGMT* promoter methylation, and have consistently proved refractory to TMZ and other chemotherapeutic agents (Jones et al., 2016). Critically, midline K27M tumors classified histologically as either grade 3 or 4 according to the WHO 2007 classification had no difference in clinical outcome within the HERBY cohort, and with the caveat of small numbers, support the current 2016 guidelines to assign all such tumors as grade 4 on the basis of their location and molecular findings.

More surprising was the poor outcome observed for patients with *H3F3A*_G34R/V mutations. A previous study reported G34R mutations to convey a better prognosis in respect of OS (HR = 0.49, p = 0.01), although this was not significant in multivariate analysis (p = 0.84) (Korshunov et al., 2015), although the present study explores this in a consistently treated and well-annotated clinical trial setting. In the HERBY study, as well as in published work (Korshunov et al., 2015, Mackay et al., 2017), the *H3F3A*_G34R/V mutation is associated with a high frequency of *MGMT* methylation. Notably, *MGMT* promoter methylation itself was not predictive in this trial cohort of all patients receiving radiochemotherapy with TMZ, again demonstrating differences with the adult disease, and questioning the continued use of protocols extrapolated from the adult setting. It is clear that histone mutations represent clearly defined entities within an umbrella HGG classification in the pediatric setting, and given their profound impact on chromatin modifications, will require therapeutic development and clinical trials distinct from histone WT cases.

Within the remaining cases of pHGG were a small proportion whose methylation profiles were more similar to other lesions. The LGG-like cases were in young patients with longer EFS, while two additional cases had methylation classifier scores strongly suggestive of recently described entities coming from the study of tumors formally diagnosed as CNS primitive neuroectodermal tumors (Sturm et al., 2016). Integration with histological features and determination of the presence of marker gene fusions events for these entities (CNS HGNET-MN1, CNS NB-FOXR2) will be key in future studies.

Although there have been several early-phase and anecdotal studies of BEV in pHGG (Benesch et al., 2008, Friedman et al., 2013, Gururangan et al., 2010, Narayana et al., 2010, Salloum et al., 2015), none have included biological information on the patients treated. Aside from CD8 immunoreactivity, we did not identify any additional molecular markers of response. In similarly designed adult studies of BEV, gene expression-defined proneural (Sandmann et al., 2015) or mesenchymal (Sulman et al., 2013) subgroups of GBM have been reported to confer a significant OS advantage. Although we have not undertaken gene expression studies across our cohort, adult and pediatric cases expressing proneural genes appear to have distinct genetic and epigenetic drivers (Sturm et al., 2012), the mesenchymal subclass is rare in children (Sturm et al., 2012), and unlike the adult studies, we observed no EFS advantage of BEV in HERBY (Grill et al., 2018).

In conclusion, integrated molecular profiling of the HERBY sample cohort has demonstrated the biological and clinicopathological diversity of the term HGG in the pediatric setting, suspected but not confirmed at the onset of the trial. While there are several distinct subgroups for which there is strong rationale for bespoke future clinical studies, a large proportion of pHGG cases continue to defy improvements in survival and lack a clear path forward. Although BEV was not associated with better outcome in this trial, the extensive biological, pathological, and radiological ancillary research programs ongoing within HERBY aim to provide an integrated assessment of disease pathogenesis and treatment response.

## STAR★Methods

### Key Resources Table

**Table:** 

REAGENT or RESOURCE	SOURCE	IDENTIFIER
**Critical Commercial Assays**		

DNeasy blood & tissue kit	Qiagen	69504
QIAmp DNA FFPE tissue kit	Qiagen	56404
RNeasy mini kit	Qiagen	74104
QIAquick PCR purification kit	Qiagen	28104
BigDye terminator v3.1 mix	Thermo Fisher	4337455
SureSelect Human All Exon capture set V6	Agilent	5190-8863
SureSelect RNA Capture, 0.5-2.9Mb	Agilent	5190-4944

**Deposited Data**		

Exome and RNA sequencing of new samples	This paper	EGA: EGAS00001002328
Illumina methylation BeadChip profiling of new samples	This paper	ArrayExpress: E-MTAB-5552
Sequencing and methylation data	This paper	cavatica.org

**Oligonucleotides**		

Primer: *H3F3A*_forward FFPE TGGCTCGTACAAAGCAGACT	This paper	N/A
Primer: *H3F3A*_reverse FFPE ATATGGATACATACAAGAGAGACT	This paper	N/A
Primer: *H3F3A* _forward FROZEN GATTTTGGGTAGACGTAATCTTCA	This paper	N/A
Primer: *H3F3A* _ reverse FROZEN TTTCCTGTTATCCATCTTTTTGTT	This paper	N/A

**Antibodies**		

MLH1	BD Pharmingen	G168-728; RRID: AB_395227
PMS2	BD Pharmingen	A16-4; RRID: AB_396410
MSH2	DIAG-BIOSYSTEMS	25D12; RRID: AB_10978033
MSH6	DIAG-BIOSYSTEMS	44; RRID: AB_1958490
H3K27M	Merck	ABE419
H3G34R	University of Nottingham	richard.grundy@nottingham.ac.uk
CD8	Dako	C8/144B; RRID: AB_2075537
CD68	Glostrup	KP1; RRID: AB_2661840

**Software and Algorithms**		

Mutation Surveyor	SoftGenetics	softgenetics.com/mutationSurveyor.php
4Peaks	Nucleobytes	nucleobytes.com/4peaks/
minfi	BioConductor	bioconductor.org/packages/release/bioc/html/minfi.html
conumee	BioConductor	bioconductor.org/packages/release/bioc/html/conumee.html
BEDtools	University of Utah	github.com/arq5x/bedtools2
DNAcopy	BioConductor	bioconductor.org/packages/release/bioc/html/DNAcopy.html
CopyNumber450kData	BioConductor	bioconductor.org/packages/release/data/experiment/html/CopyNumber450kData.html
MNP	DKFZ Heidelberg	molecularneuropathology.org/mnp
Bowtie2	Johns Hopkins University	bowtie-bio.sourceforge.net/bowtie2/index.shtml
TopHat	Johns Hopkins University	ccb.jhu.edu/software/tophat/index.shtml
cufflinks	University of Washington	ole-trapnell-lab.github.io/cufflinks/cufflinks/
DESeq2	BioConductor	bioconductor.org/packages/release/bioc/html/DESeq2.html
Gene Set Enrichment Analysis	Broad Institute	http://software.broadinstitute.org/gsea
bwa	Sanger Institute	http://bio-bwa.sourceforge.net/
Genome Analysis Toolkit	Broad Institute	oftware.broadinstitute.org/gatk/
Variant Effect predictor	Ensembl tools	ensembl.org/info/docs/variation/vep
BCBio	Harvard TH Chan	bcb.io/
ANNOVAR	Children’s Hospital of Philadelphia	annovar.openbioinformatics.org/en/latest/
ExAc	Broad Institute	exac.broadinstitute.org/
SIFT	J Craig Venter Institute	sift.jcvi.org
PolyPhen	Harvard	genetics.bwh.harvard.edu/pph2
Manta	Illumina	github.com/Illumina/manta
Oncoprinter	Memorial Sloan Kettering	cbioportal.org/oncoprinter.jsp
Circos	Michael Smith Genome Sciences Center	circos.ca
R	The Comprehensive R Archive Network	r-project.org

**Other**		

Integrated mutation, copy number, expression and methylation data	This paper and cited sources	pedcbioportal.org

### Contact for Reagent and Resource Sharing

Further information and requests for resources and reagents should be directed to and will be fulfilled by the Lead Contact, Chris Jones (chris.jones@icr.ac.uk).

### Experimental Model and Subject Details

#### Patient Samples

All patient samples were collected after signed consent to the HERBY translational research program, under full Research Ethics Committee approval at each participating center. Tumor material was available from 89 patients (out of a total of 113 providing consent) from 13 countries: France (n=27, Hospital Pour Enfants De La Timone, Marseille; Hôpital des Enfants, Toulouse; Centre Hospitalier Régional Universitaire, Tours; CHRU Rennes; CHRU Nancy; Institut Curie, Paris; CHRU Strasbourg; Cancer Research Center of Lyon; Institut Gustave Roussy, Villejuif; CHRU Clermont-Ferrand; Oscar Lambret Center, Lille; CHRU Angers), UK (n=17, Royal Victoria Infirmary, Newcastle; NHS Lothian, Edinburgh; Nottingham University Hospital, Nottingham; Great Ormond Street Hospital, London; Royal Hospital for Children, Bristol; Cambridge University Hospital, Cambridge; Leeds Teaching Hospitals, Leeds; Alder Hey Children’s Hospital, Liverpool; Royal Marsden Hospital, Sutton), Italy (n=14, Istituto Giannina Gaslini, Genoa; Istituto Nazionale Tumori, Milan), Spain (n=6, Hospital San Joan de Deu, Barcelona; Hospital Universitari i Politècnic la Fe, Valencia), Canada (n=5, Hospital for Sick Children, Toronto; Alberta Children’s Hospital, Calgary), Netherlands (n=4, Radboud University Medical Center, Nijmegen; Erasmus Medical Center, Rotterdam), Czech Republic (n=3, Faculty Hospital, Brno; Charles University Hospital, Prague), Denmark (n=3, Aarhus University Hospital, Aarhus; Rigshospitalet, Copenhagen), Hungary (n=3, Semmelweis University, Budapest), Sweden (n=3, University of Gothenburg, Gothenburg; Skåne University Hospital, Lund), Poland (n=2, Children’s Memorial Health Institute, Warsaw), Austria (n=1, Kepler Universitätsklinikum, Linz), and Belgium (n=1, Universitaire Ziekenhuizen, Leuven).

#### Pathological Review

All patients had their initial local diagnosis of HGG confirmed according to the WHO 2007 classification by a central HERBY reference neuropathologist prior to enrollment. Subsequently, all specimens were further subjected to a consensus review by the HERBY panel of five independent expert paediatric neuropathologists, who also applied the diagnostic criteria of the WHO 2016 classification.

#### Radiological Anatomical Localization

Tumor localization was determined by the HERBY panel of paediatric neuroradiologists. Tumors were assigned to lobar, basal ganglia, thalamic, non-pontine brainstem or cerebellar locations. As many of the tumors spanned more than one of these locations, post hoc radiological analysis by one of the HERBY Neuroradiologists was undertaken to determine the epicenter of the tumor origin; this was used to classify the site of origin of the tumor in each case.

### Method Details

#### Nucleic Acid Extraction

DNA was extracted from frozen tissue by homogenisation prior to following the DNeasy Blood & Tissue kit protocol (Qiagen, Crawley, UK). DNA was extracted from formalin-fixed, paraffin-embedded (FFPE) pathology blocks after manual macrodissection using the QIAamp DNA FFPE tissue kit protocol (Qiagen). Matched normal DNA was extracted from blood samples using the DNeasy Blood & Tissue kit (Qiagen, Crawley, UK). Concentrations were measured using a Qubit fluorometer (Life Technologies, Paisley, UK). RNA was extracted by following the RNeasy Mini Kit protocol (Qiagen), and quantified on a 2100 Bioanalyzer (Agilent Technologies).

#### *H3F3A* Sanger Sequencing

PCR for *H3F3A* was carried out on 89 cases using primers obtained from Life Technologies (Paisley, UK) (FFPE: for-TGGCTCGTACAAAGCAGACT; rev-ATATGGATACATACAAGAGAGACT; FROZEN: for-GATTTTGGGTAGACGTAATCTTCA; rev-TTTCCTGTTATCCATCTTTTTGTT). Sequences were analysed using Mutation Surveyor (SoftGenetics, PN, USA) and manually with 4Peaks (Nucleobytes, Aalsmeer, Netherlands). Only three cases left insufficient DNA for exome sequencing, all of which were found to harbor K27M mutations, and thus additional Sanger sequencing for genes encoding H3.1 variants were not undertaken.

#### Methylation Profiling

50-500 ng DNA was bisulphite-modified and analyzed for genome-wide methylation patterns using the Illumina HumanMethylation450 BeadChip (450k) platform at either the DKFZ or the University College London Genomics Center, according the manufacturers instructions. All samples were checked for expected and unexpected genotype matches by pairwise correlation of the 65 genotyping probes on the 450k array.

#### MGMT Promoter Methylation

To evaluate the methylation status of the *MGMT* promoter region, we used either the MGMT_STP27 logistic regression model from Illumina 450k methylation array data, or methylation-specific (MS-) PCR. For MS-PCR, 300-1500 ng DNA was sodium bisulphite-treated and PCR products analyzed on an the ABI7900HT instrument (Applied Biosystems, Foster City, CA, USA) to quantify the copy number of MGMT/ACTB (MDxHealth, Irvine, CA, USA).

#### Next-Generation Sequencing

50-500 ng DNA from 86 cases was sent for exome sequencing at the Tumor Profiling Unit (ICR, London, UK) using the Agilent SureSelectXT Human All Exon V6 platform with additional customized coverage of all histone H3 genes and the *TERT* promoter (Agilent, Santa Clara, CA, USA), and paired-end-sequenced on an Illumina HiSeq2500 (Illumina, San Diego, CA, USA) with one single patient-matched tumor/normal pair per lane where possible. The average median coverage was 426x for the frozen tumors (range 351-598x), 321x for the FFPE tumors (range 115-519x) and 163x for normal samples (range 116-464x). A customized panel of biotinylated DNA probes (NimbleGen) was developed for the detection of structural variants (translocations and duplications) and potential amplifications. The panel capture a total of 22 genes recently implicated in brain tumors (*ALK, BCOR, BRAF, c11orf95, C19MC, CIC, ETV6, FGFR1-3, FOXR2, KIAA1549, MET, MN1, MYB, MYBL1, NTRK1-3, RAF, RELA, TPM3* and *YAP1*). Library preparation was performed using 50-200 ng of genomic DNA using the HyperPlus Kit (Kapa Biosystems, Wilmington MA, USA) and SeqCap EZ adapters (Roche). Following fragmentation, DNA was end-repaired, A-tailed and indexed adapters ligated. DNA was amplified, multiplexed and hybridized using 1 μg of total pre-capture library DNA to the design of DNA baits (NimbleGen SeqCap EZ Developer library, Roche). After hybridization, capture libraries were amplified and sequencing was performed on a NextSeq500 (Illumina) with 2 x 150 bp, paired-end reads following manufacturer’s instructions. RNA from frozen tumors was sequenced on an Illumina HiSeq2500 as 100 bp paired end reads.

#### Immunohistochemistry

4 μM sections were stained by an automated Discovery XT (for H3G34R) or Benchmark XT (Ventana Medical Systems, Tucson, USA). A standard pre-treatment protocol included CC1 buffer (or CC2 for H3G34R) and then a primary antibody incubation for 32 minutes (92 minutes for MLH1, PMS2, MSH2 and MSH6) at room temperature (37°C for H3K27M and MLH1). Antibodies used were directed against MLH1 (BD Pharmingen, clone G168-728, 1/300), PMS2 (BD Pharmingen, clone A16-4, 1/150), MSH2 (DIAG-BIOSYSTEMS, clone 25D12, 1/10), MSH6 (DIAG-BIOSYSTEMS, clone 44, 1/50), H3K27M (Merck, polyclonal, 1/1000), CD8 (Dako, clone C8/144B, 1/100), CD68 (Glostrup, clone KP1, 1/400) and H3G34R (kind gift from Richard Grundy, Children’s Brain Tumor Research Center, Nottingham, UK, polyclonal; 1/150). Antibody binding was visualized with an Optiview Kit (Roche-Ventana, Tucson, USA). Diaminobenzidine-tetra-hydrochloride (DAB, Ventana) was used as the chromogen.

#### Pathological Assessment of Immune Response

CD8 immunoreactivity was assessed as the percentage of the surface area of the tumor covered by CD8^+^ cells present at a density belonging to one of four reference bins of increasing density (I0, I1, I2, I3). A further quantitative assessment of the percentage of the pathologist-defined central tumor area of CD8^+^ cells was also performed. A qualitative categorization of the tumor as a whole as either ‘inflamed’, ‘heterogeneous’ or an immune ‘desert’ was also provided. Patients in the upper quartile of central tumor area CD8 cell positivity with a heterogeneous or inflamed phenotype were classed as CD8-high. A histologically-defined assessment of tumor infiltrating lymphocytes (TILs) was carried out according to two distinct schema. Palma et al. (Palma et al., 1978) includes Category A (several perivascular cuffs of more than three layers of lymphocytes and often lymphocytes also scattered among tumor cells), Category B (three or four thin perivascular cuffs in the tumor) and Category C (no clear lymphocytes present); Rutledge et al. (Rutledge et al., 2013) includes Category 0 (absence of lymphocytes), Category 1+ (lymphocytes in less than 50% of the tumor) and Category 2+ (lymphocytes in more than 50% of the tumor).

### Quantification and Statistical Analysis

#### Sequence Analysis

Exome capture reads were aligned to the hg19 build of the human genome using bwa v0.7.5a (bio-bwa.sourceforge.net), and PCR duplicates removed with PicardTools 1.5 (pcard.sourceforge.net). Somatic single nucleotide variants were called using the Genome Analysis Tool Kit v3.3-0 based upon current Best Practices using local re-alignment around InDels, downsampling and base recalibration with variants called by the Unified Genotyper (broadinstitute.org/gatk/). Somatic variants were covered by at least 20 reads in both tumor and normal sequences and carried at least 5 ALT reads in the tumor sequence; unmatched exomes (n=3) were annotated by ExAc and ANNOVAR. Variants were annotated using the Ensembl Variant Effect Predictor v74 (ensembl.org/info/docs/variation/vep) incorporating SIFT (sift.jcvi.org) and PolyPhen (genetics.bwh.harvard.edu/pph2) predictions, COSMIC v64 (sanger.ac.uk/ genetics/CGP/cosmic/) and dbSNP build 137 (ncbi.nlm.nih.gov/sites/SNP) annotations. Copy number was obtained by calculating log_2_ ratios of tumor/normal coverage binned into exons of known Ensembl genes, smoothed using circular binary segmentation (DNAcopy, www.bioconductor.org) and processed using in-house scripts. Mutation signatures were ascertained by grouping somatic substitutions on the basis of their 3′ and 5′ bases into 96 possible trinucleotide categories (Shlien et al., 2015). NGS fusion panel alignment was performed against the human reference sequence GRCh37/Hg19. Quality control (QC), variant annotation, deduplication and metrics were generated for each sample. Manta (https://github.com/Illumina/manta) and Breakdancer (breakdancer.sourceforge.net) were used for the detection of structural variants.

#### Methylation Profiling

Methylation data from the Illumina Infinium HumanMethylation450 BeadChip was preprocessed using the minfi package in R. DNA copy number was recovered from combined intensities using the conumee package with reference to methylation profiles from normal individuals provided in the CopyNumber450kData package. We have used the Heidelberg brain tumor classifier (Capper et al., 2018) (molecularneuropathology.org) to assign subgroup scores for each tumor compared to 91 different brain tumor entities using a training set built from 2,801 tumors implemented in the MNP R package (v11b2). Simplified methylation subgroup assignments were then made to incorporate cases carrying G34R/V or K27M mutations in H3 histones, *IDH1* mutation at R132, low grade glioma-like profiles (predominantly diffuse infantile ganglioglioma and pilocytic astrocytoma) and those similar to pleomorphic xanthoastrocytoma (PXA). Low-scoring cases, or those with a high normal cell contamination were assigned to G34, K27 or IDH1 groups if the respective mutation was identified. Wild-type HGG encompassed many other methylation subgroups and were simply assigned by exclusion with the groups above. Clustering of beta values from methylation arrays was performed using the 10K probeset from the Heidelberg classifier based upon Euclidean distance with a ward algorithm. Methylation heatmaps show only the most variable probes of the classifier between simplified methylation subgroups.

#### RNAseq

RNA sequences were aligned to hg19 and organized into de-novo spliced alignments using bowtie2 and TopHat version 2.1.0 (ccb.jhu.edu/software/tophat). Fusion transcripts were detected using chimerascan version 0.4.5a filtered to remove common false positives. RNASeq raw count files were used to construct an expression matrix using Roche's internal pipeline. The expression matrix was normalized using edgeR and Voom in R (cran.rproject.org/), and a heatmap was created from the absolute gene expression data using Tibco Spotfire, as previously described (Brouwer-Visser et al., 2018). The mean expression of the signature genes was used to compare MAPK-altered to non-altered samples and statistical significance calculated using a two-tailed unpaired t-test. The mean expression was also used to correlate with CD8 positivity by IHC. Gene Set enrichment analysis was performed using the GSEA java application based upon pairwise comparisons of MAPK altered versus wild-type for curated canonical gene sets. All data are deposited in the European Genome-phenome Archive (ebi.ac.uk/ega/home) under accession number EGAS00001002328.

#### Tumor Lesion Maps

Pre-surgery tumor volume regions of interest (ROIs) were drawn on T2-weighted/Fluid-attenuated inversion recovery (FLAIR) magnetic resonance (MR) images by an experienced paediatric neuroradiologist (TJ). The images and corresponding ROIs were affinely registered, using FSL (Jenkinson et al., 2002), to a paediatric template (Left-Right Symmetric, 7.5–13.5 years old) from the Montreal Neurological Institute (http://www.bic.mni.mcgill.ca/ServicesAtlases/NIHPD-obj1) (Fonov et al., 2011). A further manual correction step was performed to limit tumor mass effects. Once registered to a common space, overall tumor lesion overlap maps were created using MRIcron (Rorden et al., 2007).

#### Statistical Analysis

Statistical analysis was carried out using R 3.3.0 (www.r-project.org) and GraphPad Prism 7. Categorical comparisons of counts were carried out using Fishers exact test, comparisons between groups of continuous variables employed Student’s t-test or ANOVA. Univariate differences in survival were analysed by the Kaplan-Meier method and significance determined by the log-rank test. Exploratory Cox regression analyses were conducted to assess the impact of molecular prognostic and predictive factors. Confirmation of proportional hazards was assessed by calculating Schoenfeld residuals. All tests were two-sided and a p value of less than 0.05 was considered significant.

### Data and Software Availability

All newly generated data have been deposited in the European Genome-phenome Archive (www.ebi.ac.uk/ega) with accession number EGAS00001002328 (sequencing) or ArrayExpress (www.ebi.ac.uk/arrayexpress/) with accession number E-MTAB-5552 (450k methylation).

### Additional Resources

Curated gene-level copy number, mutation data and RNAseq data are provided as part of the paediatric-specific implementation of the cBioPortal genomic data visualization portal (pedcbioportal.org). Raw data files are also made available through the Cavatica NIH-integrated cloud platform (cavatica.org).

## Supplemental Information

Document S1. Figures S1–S7

Table S1. Summary of Clinical and Molecular Data, Related to Figure 1

Table S2. Methylation Subclassifications, Related to Figure 2

Table S3. Sequencing Results, Related to Figure 3

Table S4. Immune Profiling, Related to Figure 5

Table S5. Gene Set Enrichment Analysis, Related to Figure 7 NAME, names of curated canonical datasets; SIZE, number of genes in gene set; ES, the enrichment score; NES, normalized enrichment score; NOM p val, the nominal p value; FDR q-val, the false discovery rate q value; FWER p val, the family-wise error rate p value; RANK AT MAX, the position in the ranked list at which the maximum enrichment score occurred.

Document S2. Article plus Supplemental Information
