# Supplementary material for: Molecular, Pathological, Radiological, and Immune Profiling of Non-brainstem Pediatric High-Grade Glioma from the HERBY Phase II Randomized Trial
Source: Cancer Cell. 2018 May 14;33(5):829–842.e5. doi: 10.1016/j.ccell.2018.04.004 (PMC5956280; doi:10.1016/j.ccell.2018.04.004)
Supplement: Document S1. Figures S1–S7 [file mmc1.pdf]

Cancer Cell, Volume *33*

## Supplemental Information

# Molecular, Pathological, Radiological, and Immune Profiling of Non-brainstem Pediatric High-Grade Glioma from the HERBY Phase II Randomized Trial

**Alan Mackay, Anna Burford, Valeria Molinari, David T.W. Jones, Elisa Izquierdo, Jurriaan Brouwer-Visser, Felice Giangaspero, Christine Haberler, Torsten Pietsch, Thomas S. Jacques, Dominique Figarella-Branger, Daniel Rodriguez, Paul S. Morgan, Pichai Raman, Angela J. Waanders, Adam C. Resnick, Maura Massimino, Maria Luisa Garrè, Helen Smith, David Capper, Stefan M. Pfister, Thomas Würdinger, Rachel Tam, Josep Garcia, Meghna Das Thakur, Gilles Vassal, Jacques Grill, Tim Jaspan, Pascale Varlet, and Chris Jones**

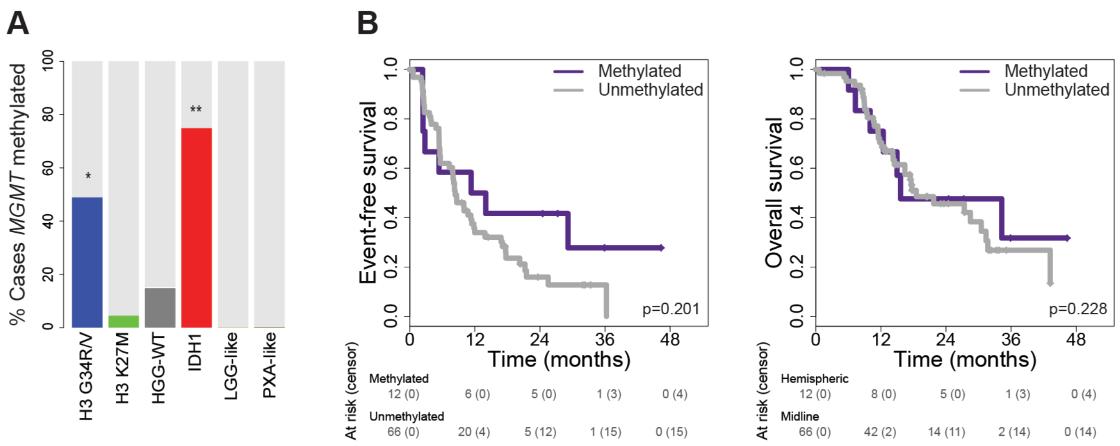


**Figure S1** (related to Figure 2) – *MGMT* promoter methylation. (A) Barplots of number of cases with methylated *MGMT* promoter, subdivided by methylation subgroup. (B) Kaplan-Meier plot of event-free and overall survival of cases (y axis) separated by *MGMT* status, time given in months (x axis) and p value calculated by the log-rank test.

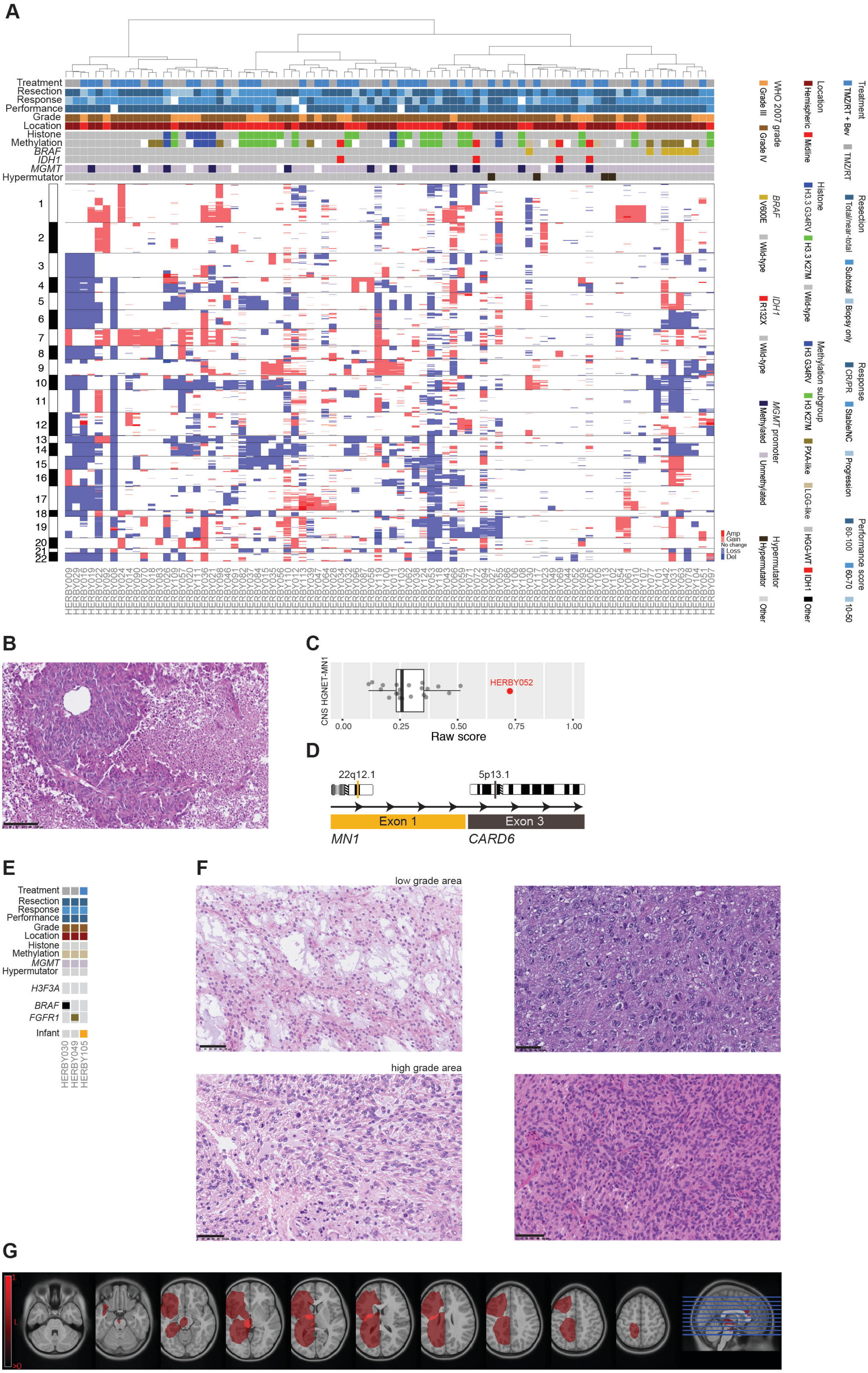
A
Treatment
Resection
Response
Performance
Grade
Location
Histone
Methylation
BRAF
IDH1
MGMT
Hypermutator
Amp
Gain
No change
Loss
Del
Treatment
TMZ/RT + Bev
TMZ/RT
Resection
Total/near-total
Subtotal
Biopsy only
Response
CR/PR
Stable/NC
Progression
Performance score
80-100
60-70
10-50
Location
Hemispheric
Midline
Histone
H3.3 G34R/V
H3.3 K27M
Wild-type
Methylation subgroup
H3 G34R/V
H3 K27M
PXA-like
LGG-like
HGG-WT
IDH1
Other
WHO 2007 grade
Grade III
Grade IV
BRAF
V600E
Wild-type
IDH1
R132X
Wild-type
MGMT promoter
Methylated
Unmethylated
Hypermutator
Hypermutator
Other
B
C
CNS HGNET-MN1
HERBY052
0.00
0.25
0.50
0.75
1.00
Raw score
D
22q12.1
5p13.1
Exon 1
Exon 3
MN1
CARD6
E
Treatment
Resection
Response
Performance
Grade
Location
Histone
Methylation
MGMT
Hypermutator
H3F3A
BRAF
FGFR1
Infant
HERBY030
HERBY049
HERBY105
F
low grade area
high grade area
G

**Figure S2** (related to Figure 3) – DNA copy number profiling and non-HGG-like entities. (A) Heatmap representation of segmented DNA copy number for 86 samples derived from exome coverage data (dark red, amplification; red, gain; dark blue, deletion; blue, loss). Samples are arranged in columns clustered by gene-level data across the whole genome. Clinicopathological and molecular annotations are provided as bars according to the included key. CR/PR = complete response or partial response; Stable/NC = stable disease or no change. (B-D) CNS HGNET-MN1. Haematoxylin and eosin staining (B) of the case most closely resembling CNS HGNET-MN1 (HERBY052), as demonstrated by a boxplot of reference methylation classifier scores (C), and the presence of an *MN1:CARD6* gene fusion by capture panel sequencing (D). Scale bar = 100 µm. (E) Integrated annotation of somatic mutations and DNA copy number changes in 3 samples classifying as LGG-like. Clinicopathological and molecular annotations are provided as bars according to the included key in Figure S1. (F) Haematoxylin and eosin staining of the three cases, all histologically classified as glioblastoma – (left) the presence of both low- (upper panel) and high grade (lower panel) areas of the tumor harboring *BRAF*_V600E mutation (HERBY049); (top right) case harboring an intragenic *FGFR1* duplication (HERBY030; both previous cases classifying as pilocytic astrocytoma); (bottom left) a cased from the infant cohort, with a methylation profile most closely resembling desmoplastic infantile ganglioglioma (HERBY105). Scale bar = 50 µm. (G) Radiological tumor lesion map of LGG-like cases. Brighter colored pixels indicate a higher probability of tumor incidence.

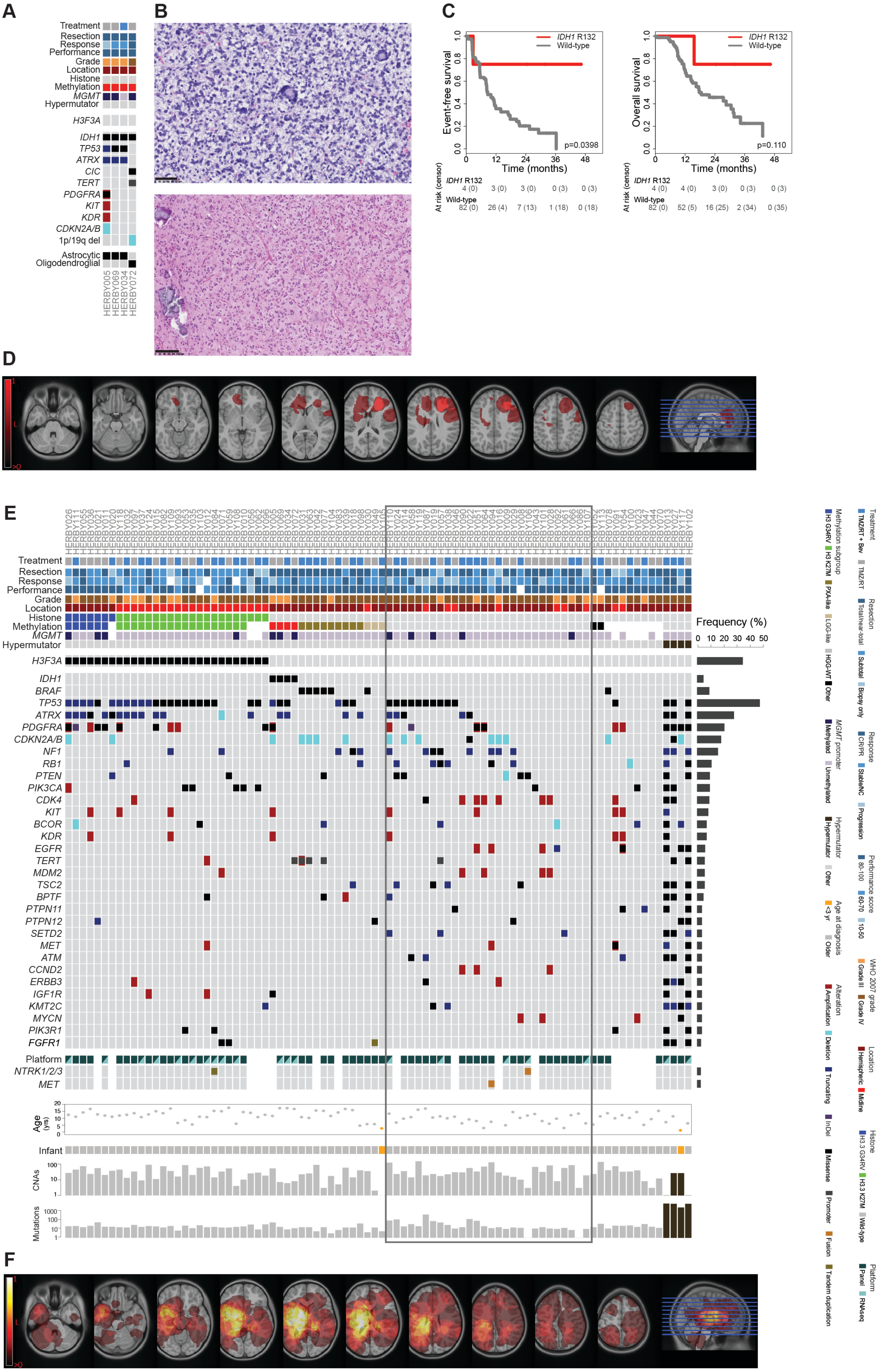
A
Treatment
Resection
Response
Performance
Grade
Location
Histone
Methylation
MGMT
Hypermutator
H3F3A
IDH1
TP53
ATRX
CIC
TERT
PDGFRA
KIT
KDR
CDKN2A/B
1p/19q del
Astrocytic
Oligodendroglial
HERBY005
HERBY069
HERBY034
HERBY072
B
C
IDH1 R132
Wild-type
Event-free survival
Overall survival
Time (months)
p=0.0398
p=0.110
At risk (censor)
D
E
Frequency (%)
Platform
NTRK1/2/3
MET
Age (yrs)
Infant
CNAs
Mutations
F
Treatment
TMZ/RT + Bev
TMZ/RT
Resection
Total/near-total
Subtotal
Biopsy only
Response
CR/PR
Stable/NC
Progression
Performance score
80-100
60-70
10-50
WHO 2007 grade
Grade III
Grade IV
Location
Hemispheric
Midline
Histone
H3.3 G34R/V
H3.3 K27M
Wild-type
Platform
Panel
RNAseq
Methylation subgroup
H3 G34R/V
H3 K27M
PXA-like
LGG-like
HGG-WT
Other
MGMT promoter
Methylated
Unmethylated
Hypermutator
Hypermutator
Other
Age at diagnosis
<3 yr
Older
Alteration
Amplification
Deletion
Truncating
InDel
Missense
Promoter
Fusion
Tandem duplication

**Figure S3** (related to Figure 3) – *IDH1* mutant and *H3F3A*/*IDH1*/*BRAF* wild-type tumors. (A) Integrated annotation of somatic mutations and DNA copy number changes in 4 samples with *IDH1*_R132 mutation. Clinicopathological and molecular annotations are provided as bars according to the included key in Figure S1. (B) Haematoxylin and eosin staining of cases showing astrocytic (top, HERBY005) and oligodendroglial (bottom, HERBY072) histological features. Scale bar = 50 μm (top) and 100 μm (bottom). (C) Kaplan-Meier plot of event-free and overall survival of cases (y axis) separated by *IDH1*_R132 status, time given in months (x axis) and p value calculated by the log-rank test. (D) Radiological tumor lesion map of *IDH1* cases. Brighter colored pixels indicate a higher probability of tumor incidence. (E) Oncoprint representation of an integrated annotation of somatic mutations and DNA copy number changes for the 30 most frequently altered genes in 86 samples ($n \geq 3$, frequency barplot on the right, excluding hypermutator cases), ordered by histone and methylation subgroups. Selected common fusion events are also shown where available. Samples are arranged in columns with genes labelled along rows. Barplots are provided on a $\log_{10}$ scale for numbers of copy number aberrations and somatic mutations per case. Clinicopathological and molecular annotations are provided as bars according to the included key. CR/PR = complete response or partial response; Stable/NC = stable disease or no change. The annotated box highlights *H3F3A*/*IDH1*/*BRAF* wild-type 'HGG-WT' tumors not otherwise assigned to a subgroup. (F) Radiological tumor lesion map of HGG-WT cases. Brighter colored pixels indicate a higher probability of tumor incidence.

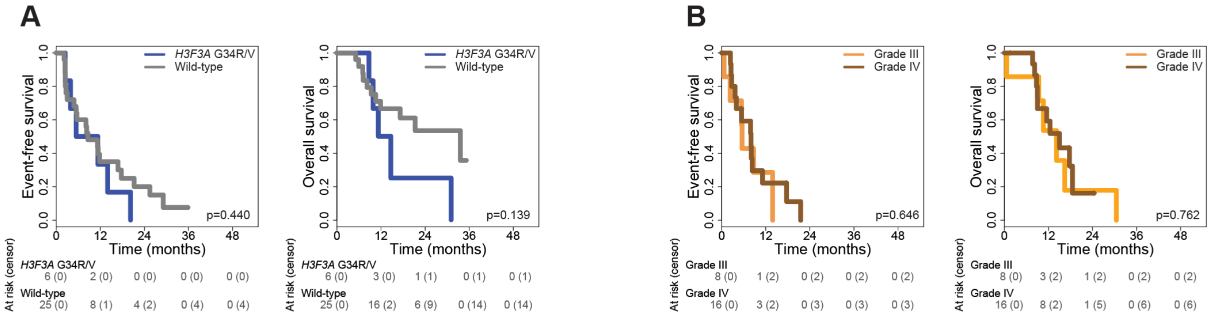


**Figure S4** (related to Figure 4) – *H3F3A* mutant subgroups. (A) Kaplan-Meier plot of event-free and overall survival (y axis) of 31 cerebral hemispheric cases, excluding those classified by methylation profiling as IDH1, PXA-like or LGG-like, separated by *H3F3A* status. Time is given in months (x axis) and p value calculated by the log-rank test. (B) Kaplan-Meier plot of event-free and overall survival (y axis) of 24 midline *H3F3A*_K27M cases, separated by WHO grade. Time is given in months (x axis) and p value calculated by the log-rank test.

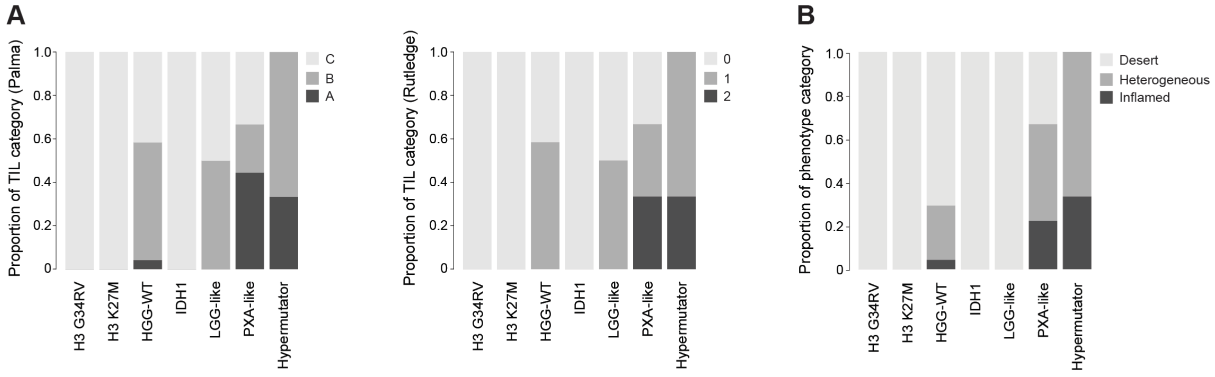


**Figure S5** (related to Figure 5) – Immune profiling. (A) Barplot showing relative proportions of tumor-infiltrating lymphocytes categorized according to two schema (Palma and Rutledge), of 72 cases split by pHGG subgroups. (B) Barplot showing relative proportions of histologically defined immune phenotype of 72 cases, split by pHGG subgroups.

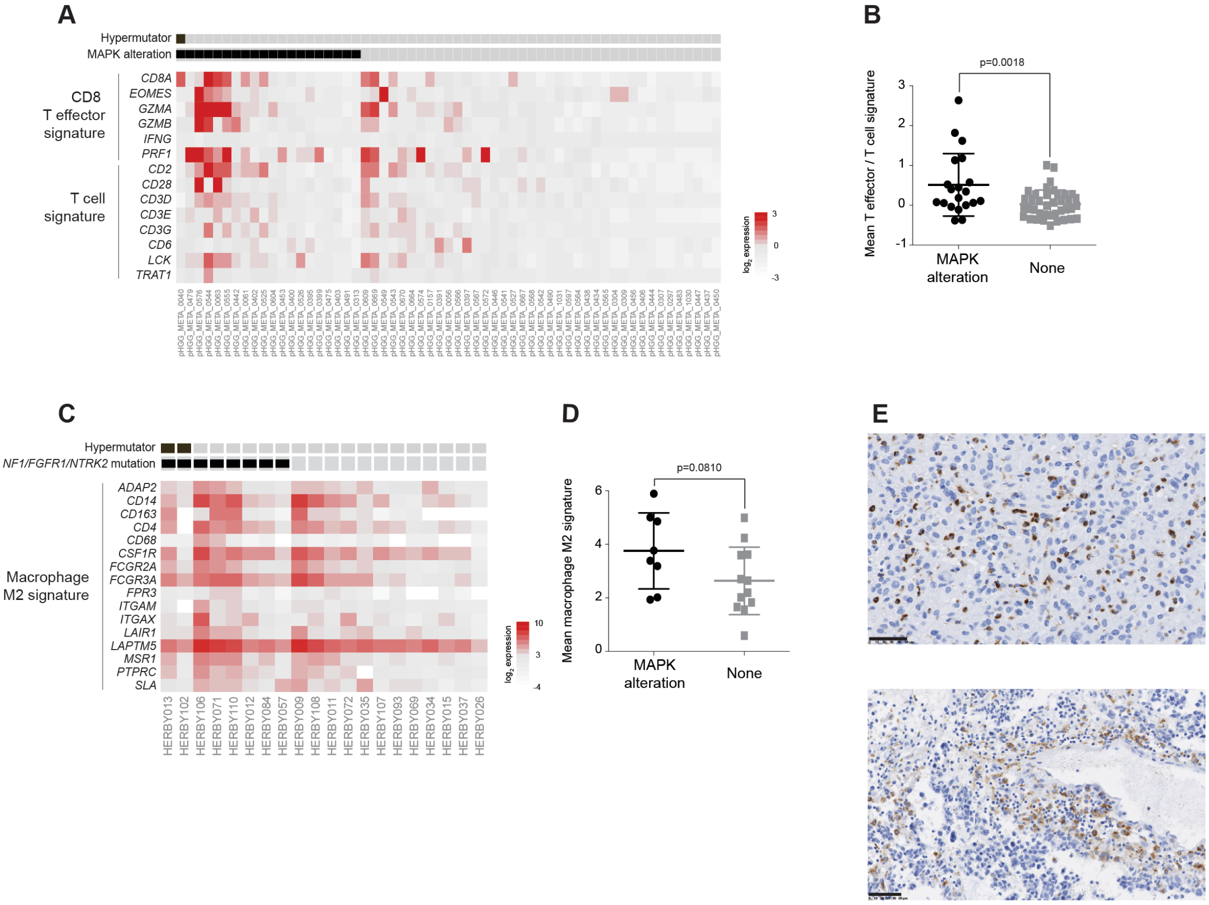
A
Hypermutator
MAPK alteration
CD8
T effector
signature
T cell
signature
CD8A
EOMES
GZMA
GZMB
IFNG
PRF1
CD2
CD28
CD3D
CD3E
CD3G
CD6
LCK
TRAT1
log2 expression
3
0
-3
B
p=0.0018
Mean T effector / T cell signature
3
2
1
0
-1
MAPK alteration
None
C
Hypermutator
NF1/FGFR1/NTRK2 mutation
Macrophage
M2 signature
ADAP2
CD14
CD163
CD4
CD68
CSF1R
FCGR2A
FCGR3A
FPR3
ITGAM
ITGAX
LAIR1
LAPTM5
MSR1
PTPRC
SLA
log2 expression
10
3
-4
HERBY013
HERBY102
HERBY106
HERBY071
HERBY110
HERBY012
HERBY084
HERBY057
HERBY009
HERBY108
HERBY011
HERBY072
HERBY035
HERBY107
HERBY093
HERBY069
HERBY034
HERBY015
HERBY037
HERBY026
D
p=0.0810
Mean macrophage M2 signature
6
4
2
0
MAPK alteration
None
E

**Figure S6** (related to Figure 7) – CD8 and CD68 signatures. (A) Gene expression signatures for CD8 T effector and T cells plotted as a heatmap from combined gene expression data of non-brainstem high-grade glioma in n=59 patients aged 3-18 years from Mackay *et al.*, 2017. Hypermutator cases and those with MAPK alterations are annotated. (B) Boxplot of T effector / T cell gene expression values in MAPK altered samples compared to those without. Horizontal bar represents the mean, error bars the standard deviation. (C) Gene expression signatures for M2 macrophages plotted as a heatmap from 20 cases with RNAseq data. Hypermutator cases and those with MAPK alterations are annotated. (D) Boxplot of M2 macrophage cell gene expression values in MAPK altered samples compared to those without. Horizontal bar represents the mean, error bars the standard deviation. (E) Immunohistochemistry directed against CD68, showing positive cells in perivascular areas associated with lymphocytes (top, HERBY104, hypermutator) and more diffusely mixed with tumor cells (bottom, HERBY102, *BRAF*_V600E). Scale bar = 50 µm.

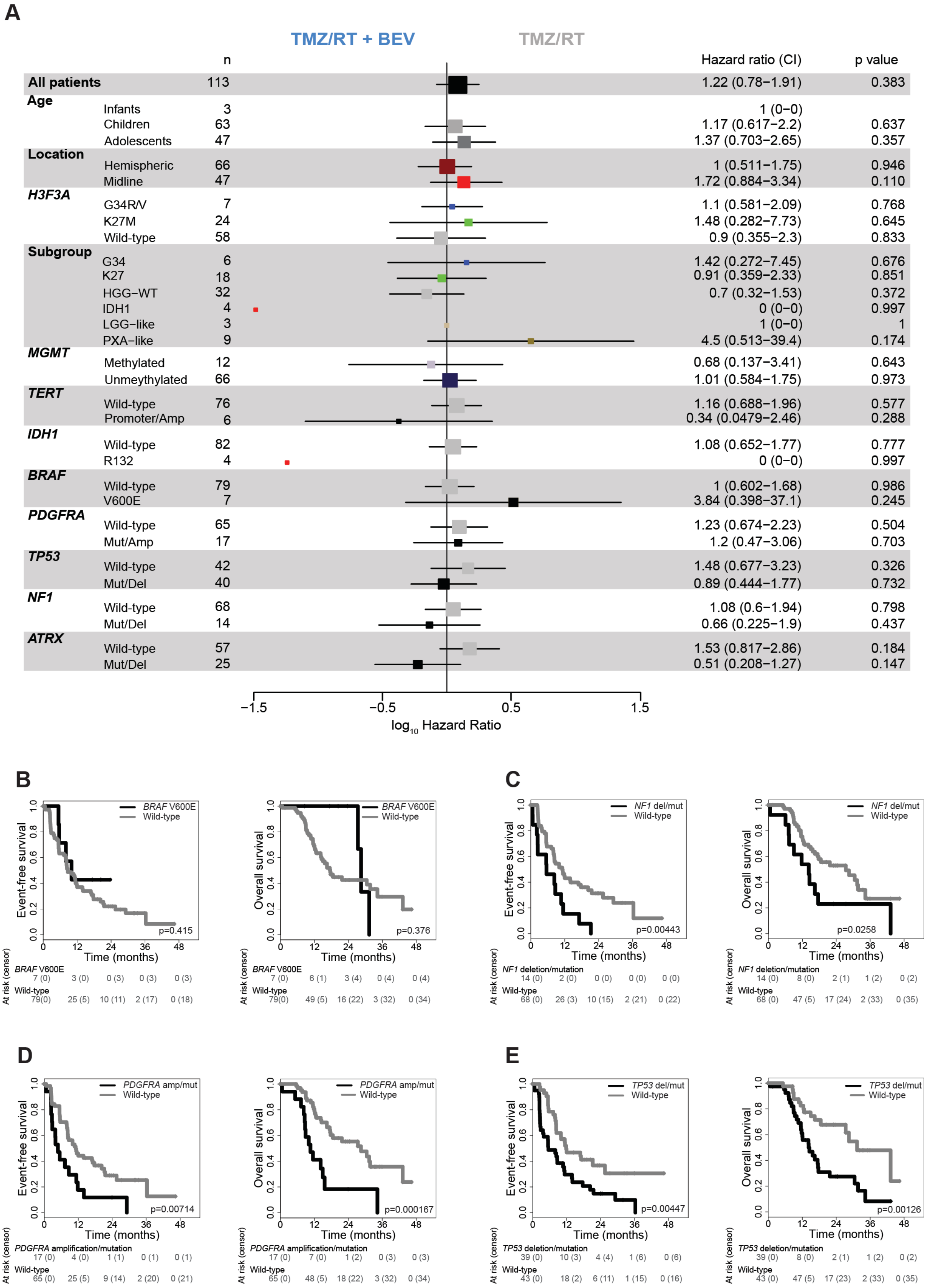
A
TMZ/RT + BEV
TMZ/RT
n
Hazard ratio (CI)
p value
All patients 113 1.22 (0.78−1.91) 0.383
Age
Infants 3 1 (0−0)
Children 63 1.17 (0.617−2.2) 0.637
Adolescents 47 1.37 (0.703−2.65) 0.357
Location
Hemispheric 66 1 (0.511−1.75) 0.946
Midline 47 1.72 (0.884−3.34) 0.110
H3F3A
G34R/V 7 1.1 (0.581−2.09) 0.768
K27M 24 1.48 (0.282−7.73) 0.645
Wild-type 58 0.9 (0.355−2.3) 0.833
Subgroup
G34 6 1.42 (0.272−7.45) 0.676
K27 18 0.91 (0.359−2.33) 0.851
HGG−WT 32 0.7 (0.32−1.53) 0.372
IDH1 4 0 (0−0) 0.997
LGG−like 3 1 (0−0) 1
PXA−like 9 4.5 (0.513−39.4) 0.174
MGMT
Methylated 12 0.68 (0.137−3.41) 0.643
Unmeythylated 66 1.01 (0.584−1.75) 0.973
TERT
Wild-type 76 1.16 (0.688−1.96) 0.577
Promoter/Amp 6 0.34 (0.0479−2.46) 0.288
IDH1
Wild-type 82 1.08 (0.652−1.77) 0.777
R132 4 0 (0−0) 0.997
BRAF
Wild-type 79 1 (0.602−1.68) 0.986
V600E 7 3.84 (0.398−37.1) 0.245
PDGFRA
Wild-type 65 1.23 (0.674−2.23) 0.504
Mut/Amp 17 1.2 (0.47−3.06) 0.703
TP53
Wild-type 42 1.48 (0.677−3.23) 0.326
Mut/Del 40 0.89 (0.444−1.77) 0.732
NF1
Wild-type 68 1.08 (0.6−1.94) 0.798
Mut/Del 14 0.66 (0.225−1.9) 0.437
ATRX
Wild-type 57 1.53 (0.817−2.86) 0.184
Mut/Del 25 0.51 (0.208−1.27) 0.147
−1.5 −0.5 0.5 1.5
log10 Hazard Ratio
B
Event-free survival
BRAF V600E
Wild-type
p=0.415
Time (months)
At risk (censor)
BRAF V600E 7 (0) 3 (0) 0 (3) 0 (3) 0 (3)
Wild-type 79(0) 25 (5) 10 (11) 2 (17) 0 (18)
Overall survival
BRAF V600E
Wild-type
p=0.376
Time (months)
At risk (censor)
BRAF V600E 7 (0) 6 (1) 3 (4) 0 (4) 0 (4)
Wild-type 79(0) 49 (5) 16 (22) 3 (32) 0 (34)
C
Event-free survival
NF1 del/mut
Wild-type
p=0.00443
Time (months)
At risk (censor)
NF1 deletion/mutation 14 (0) 2 (0) 0 (0) 0 (0) 0 (0)
Wild-type 68 (0) 26 (3) 10 (15) 2 (21) 0 (22)
Overall survival
NF1 del/mut
Wild-type
p=0.0258
Time (months)
At risk (censor)
NF1 deletion/mutation 14 (0) 8 (0) 2 (1) 1 (2) 0 (2)
Wild-type 68 (0) 47 (5) 17 (24) 2 (33) 0 (35)
D
Event-free survival
PDGFRA amp/mut
Wild-type
p=0.00714
Time (months)
At risk (censor)
PDGFRA amplification/mutation 17 (0) 4 (0) 1 (1) 0 (1) 0 (1)
Wild-type 65 (0) 25 (5) 9 (14) 2 (20) 0 (21)
Overall survival
PDGFRA amp/mut
Wild-type
p=0.000167
Time (months)
At risk (censor)
PDGFRA amplification/mutation 17 (0) 7 (0) 1 (2) 0 (3) 0 (3)
Wild-type 65 (0) 48 (5) 18 (22) 3 (32) 0 (34)
E
Event-free survival
TP53 del/mut
Wild-type
p=0.00447
Time (months)
At risk (censor)
TP53 deletion/mutation 39 (0) 10 (3) 4 (4) 1 (6) 0 (6)
Wild-type 43 (0) 18 (2) 6 (11) 1 (15) 0 (16)
Overall survival
TP53 del/mut
Wild-type
p=0.00126
Time (months)
At risk (censor)
TP53 deletion/mutation 39 (0) 8 (0) 2 (1) 1 (2) 0 (2)
Wild-type 43 (0) 47 (5) 17 (23) 2 (33) 0 (35)

**Figure S7** (related to Figure 7) – Exploratory biomarker analysis. (A) Hazard ratio plot for a univariate Cox regression analysis on a variety of molecular subgroups and alterations in respect of event-free survival. $Log_2$ hazard ratios less than zero indicate a better response to TMZ/RT plus BEV, ratios greater than zero a better response to TMZ/RT alone. Median (box) and 95% confidence intervals (whiskers) are plotted, with size of box proportion to sample size on an indicated category of tumors. (B-E) Kaplan-Meier plot of event-free and overall survival of cases (y axis) separated by *BRAF*_V600E (B), *NF1* (C), *PDGFRA* (D) and *TP53* (E) status, time given in months (x axis) and p value calculated by the log-rank test.
